# Identification of a novel quinoline-based DNA demethylating compound highly potent in cancer cells

**DOI:** 10.1186/s13148-019-0663-8

**Published:** 2019-05-06

**Authors:** Clemens Zwergel, Michael Schnekenburger, Federica Sarno, Cecilia Battistelli, Maria Cristina Manara, Giulia Stazi, Roberta Mazzone, Rossella Fioravanti, Christina Gros, Frédéric Ausseil, Cristina Florean, Angela Nebbioso, Raffaele Strippoli, Toshikazu Ushijima, Katia Scotlandi, Marco Tripodi, Paola B. Arimondo, Lucia Altucci, Marc Diederich, Antonello Mai, Sergio Valente

**Affiliations:** 1 Department of Chemistry and Technologies of Drugs, Sapienza University of Rome, P.le A. Moro 5, 00185 Rome, Italy; 2 Laboratoire de Biologie Moléculaire et Cellulaire du Cancer, Hôpital Kirchberg, 9 rue Edward Steichen, L-2540 Luxembourg City, Luxembourg; 3 Department of Medicine of Precision, University of Studi della Campania Luigi Vanvitelli, Vico L. De Crecchio 7, 80138 Naples, Italy; 4 Department of Molecular Medicine, Sapienza University of Rome, Viale Regina Elena 324, 00161 Rome, Italy; 5 Laboratory of Experimental Oncology, IRCCS - Istituto Ortopedico Rizzoli, via di Barbiano, 1/10, Bologna, 40136 Italy; 6 Center for High-Throughput Chemical Biology, Simon Fraser University, 8888 University Drive, Burnaby, BC V5A 1S6 Canada; 7 Pierre Fabre Laboratories, 3 Avenue Hubert Curien, Toulouse, 31100 France; 8 National Institute for Infectious Diseases L. Spallanzani, IRCCS, Via Portuense, 292, Rome, 00149 Italy; 9 Division of Epigenomics, National Cancer Center Research Institute, 5-1-1 Tsukiji, Chuo-ku, Tokyo, 104-0045 Japan; 10 Pasteur Institute, Cenci-Bolognetti Foundation, Sapienza University of Rome, P.le A. Moro 5, 00185 Rome, Italy; 11 Epigenetic Chemical Biology, Institut Pasteur, CNRS UMR3523, 28 rue du Docteur Roux, Paris, 75724 France; 12 Department of Pharmacy, Research Institute of Pharmaceutical Sciences, College of Pharmacy, Seoul National University, 1 Gwanak-ro, Gwanak-gu, 08826 Korea

**Keywords:** Epigenetics, Cancer, DNMT inhibitor, Quinoline-based compound, Gene expression

## Abstract

**Background:**

DNA methyltransferases (DNMTs) are epigenetic enzymes involved in embryonic development, cell differentiation, epithelial to mesenchymal transition, and control of gene expression, whose overexpression or enhanced catalytic activity has been widely reported in cancer initiation and progression. To date, two DNMT inhibitors (DNMTi), 5-azacytidine (5-AZA) and 5-aza-2′-deoxycytidine (DAC), are approved for the treatment of myelodysplastic syndromes and acute myeloid leukemia. Nevertheless, they are chemically instable and quite toxic for healthy cells; thus, the discovery of novel DNMTi is urgent.

**Results:**

Here, we report the identification of a new quinoline-based molecule, MC3353, as a non-nucleoside inhibitor and downregulator of DNMT. This compound was able, in promoter demethylating assays, to induce enhanced green fluorescence protein (EGFP) gene expression in HCT116 cells and transcription in a cytomegalovirus (CMV) promoter-driven luciferase reporter system in KG-1 cells. Moreover, MC3353 displayed a strong antiproliferative activity when tested on HCT116 colon cancer cells after 48 h of treatment at 0.5 μM. At higher doses, this compound provided a cytotoxic effect in double DNMT knockout HCT116 cells. MC3353 was also screened on a different panel of cancer cells (KG-1 and U-937 acute myeloid leukemia, RAJI Burkitt’s lymphoma, PC-3 prostate cancer, and MDA-MB-231 breast cancer), where it arrested cell proliferation and reduced viability after 48 h of treatment with IC_50_ values ranging from 0.3 to 0.9 μM. Compared to healthy cell models, MC3353 induced apoptosis (e.g., U-937 and KG-1 cells) or necrosis (e.g., RAJI cells) at lower concentrations. Importantly, together with the main DNMT3A enzyme inhibition, MC3353 was also able to downregulate the DNMT3A protein level in selected HCT116 and PC-3 cell lines. Additionally, this compound provided impairment of the epithelial-to-mesenchymal transition (EMT) by inducing E-cadherin while reducing matrix metalloproteinase (MMP2) mRNA and protein levels in PC-3 and HCT116 cells. Last, tested on a panel of primary osteosarcoma cell lines, MC3353 markedly inhibited cell growth with low single-digit micromolar IC_50_ ranging from 1.1 to 2.4 μM. Interestingly, in Saos-2 osteosarcoma cells, MC3353 induced both expression of genes and mineralized the matrix as evidence of osteosarcoma to osteoblast differentiation.

**Conclusions:**

The present work describes MC3353 as a novel DNMTi displaying a stronger in cell demethylating ability than both 5-AZA and DAC, providing re-activation of the silenced ubiquitin C-terminal hydrolase L1 (UCHL1) gene. MC3353 displayed dose- and time-dependent antiproliferative activity in several cancer cell types, inducing cell death and affecting EMT through E-cadherin and MMP2 modulation. In addition, this compound proved efficacy even in primary osteosarcoma cell models, through the modulation of genes involved in osteoblast differentiation.

**Electronic supplementary material:**

The online version of this article (10.1186/s13148-019-0663-8) contains supplementary material, which is available to authorized users.

## Introduction

Epigenetics studies the changes in gene expression not caused by alterations in the DNA base sequence and leading to a heritable and stable phenotype [1–3]. The epigenetic machinery is regulated by chromatin modifications involving histone and/or DNA modifications and nucleosome positioning and by non-coding RNAs [4]. In humans, a main epigenetic modification is DNA methylation, described as a stable epigenetic mark occurring at the C-5 position of cytosine residues in DNA regions called CpG islands. The reaction is catalyzed by DNA methyltransferases (DNMTs, namely DNMT1, DNMT3A, and DNMT3B), which use hemimethylated or unmethylated DNA as the substrate and *S*-adenosyl-l-methionine (AdoMet) as the methyl donor co-substrate [5, 6].

**Fig. 1:**
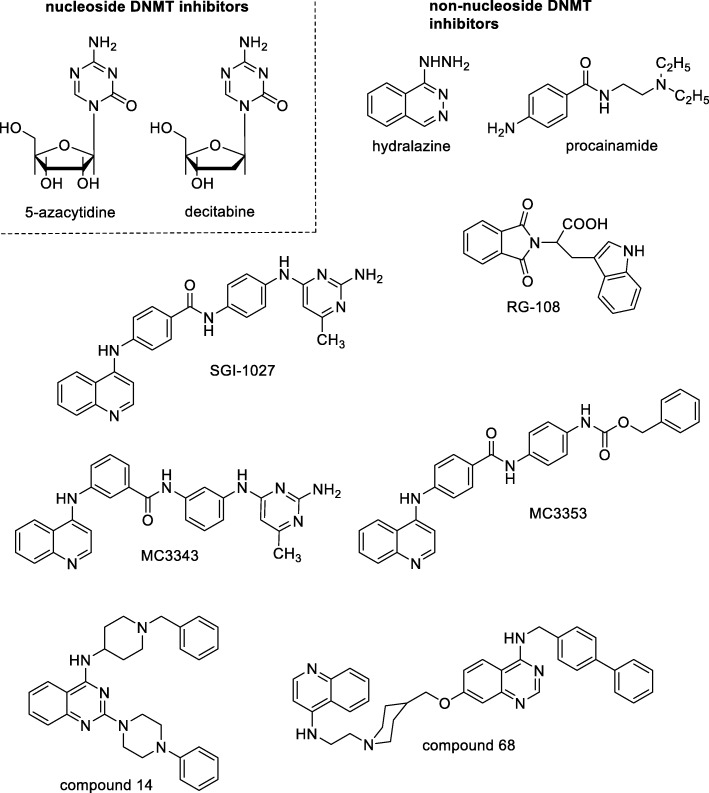
Chemical structures of known nucleoside and non-nucleoside DNMT inhibitors

DNA methylation is essential for crucial processes such as embryonic development or differentiation [5, 7]. In cancer, aberrant DNA methylation patterns have been extensively described, leading to CpG island hypermethylation together with global hypomethylation and subsequent inactivation of tumor suppressor genes (TSGs) and genomic instability [5, 7–9]. Genetic or pharmacological DNMT1 inhibition has been reported to be sufficient to lead to TSG re-expression and cell growth arrest in a variety of cancer cells in vitro, such as lung, esophagus, stomach, breast, cervix, brain, head, and neck cancer cells [10–14]. DNMT inhibitors (DNMTi) are currently classified into nucleoside analogs including 5-azacytidine (5-AZA) and 5-aza-2′-deoxycytidine (DAC; decitabine), approved in 2004 and 2006, respectively, by US FDA for the treatment of myelodysplasia and other hematological malignancies, and non-nucleoside analogs, obtained by natural sources, repositioning of old drugs (for example, hydralazine and procainamide [13–15]) or by identification of new chemical entities (RG-108 [16] and its benzoylproline analog [17], nitrophenyl-chromenones and -chromanones [18], SGI-1027 [19] and its regioisomer MC3343 [20–22], quinazoline derivatives such as compound 14 [23], and compound 68 [24]) (Fig. 1).

The two approved nucleoside DNMTi suffer from poor bioavailability, chemical instability, and adverse side effects, while non-nucleoside DNMTi are often not specific, scarcely potent, and with an unknown mechanism of action [5, 12–14]. Among the latter, SGI-1027 is the most reliable compound, since it shows a significant effect against DNMT1 and DNMT3A at a low micromolar level and displays arrest of proliferation and TSG reactivation in cancer cells [19]. Previously, we performed chemical manipulations on the SGI-1027 structure by preparing all of its regioisomers as well as some truncated and symmetric bis-quinoline and bis-pyrimidine analogs [20]. From this study, the *N*-(3-(2-amino-6-methylpyrimidin-4-ylamino)phenyl)-3-(quinolin-4-ylamino)benzamide MC3343 (Fig. 1) emerged as a novel hit compound showing higher potency and selectivity than SGI-1027 with respect to other AdoMet-dependent histone/protein methyltransferases [20] and a different mechanism of action [21]. In a panel of cancer cells, MC3343 showed similar antiproliferative activities as SGI-1027 but reduced toxicity [20], even displaying inhibition of proliferation and induction of cytodifferentiation in medulloblastoma cancer stem cells [20]. Furthermore, MC3343 also impaired tumor proliferation of osteosarcoma cells by blocking the cell cycle in G1 or G2/M phases and induced osteoblastic differentiation through the specific re-expression of genes regulating this physiological process. MC3343 displayed these biological effects even in vivo against a patient-derived xenograft [22].

Prompted by these results, we designed a novel analog of SGI-1027, MC3353, by replacing the 4-methyl-2, 6-diaminopyrimidine moiety of the template with a benzyl carbamate function (Fig. 1). This new compound was tested against human DNMT1 and DNMT3A in vitro. Then, it was tested in a stable cellular luciferase cytomegalovirus (CMV) reporter system (CMV-luc) integrated in human leukemia KG-1 cells in which the luciferase gene is under the control of the methylated CMV promoter, as well as in HCT116 human colon cancer cells to detect its ubiquitin C-terminal hydrolase L1 (UCHL1) promoter demethylating ability, revealed by enhanced green fluorescent protein (EGFP) reporter gene expression. Moreover, MC3353 was tested in a panel of cancer cells (HCT116 colon cancer, KG-1, U-937 acute myeloid leukemia, MDA-MB-231 breast cancer, RAJI Burkitt’s lymphoma, and PC-3 prostate cancer) to determine its effects on cell proliferation, viability, and apoptosis/necrosis induction. SGI-1027, as well as DAC and/or 5-AZA, were used as reference drugs in all assays. In addition, the effect of MC3353 on epithelial-to-mesenchymal transition (EMT), a process controlling cellular invasiveness, was investigated in PC-3 and HCT116 cells. The recent results provided by MC3343 treatment of osteosarcoma cell and mouse models [22] suggested us to also test MC3353 in this cellular context. Accordingly, we evaluated the effect of MC3353 on the proliferation of patient-derived Saos-2, U-2OS, MG63, IOR/OS9, IOR/OS20, IOR/SARG, and patient-derived xenograft PDX-OS#1-C4 cells. Finally, we tested the impact of MC3353 on the expression of three genes [collagen type I alpha 2 chain (COL1A2), alkaline phosphatase (ALP), and osteocalcin (OCN)] involved in osteosarcoma differentiation processes as well as on matrix mineralization in Saos-2 cells.

## Materials and methods

### Chemistry

Melting points were determined on a Büchi 530 melting point apparatus and are uncorrected. ^1^H nuclear magnetic resonance (NMR) and ^13^C NMR spectra were recorded at 400 MHz on a Bruker AC 400 spectrometer; chemical shifts are reported in *δ* (ppm) units relative to the internal reference tetramethylsilane (Me_4_Si). EIMS spectra were recorded with a Fisons Trio 1000 spectrometer; only molecular ions (M^+^) and base peaks are given. All compounds were routinely checked by thin layer chromatography (TLC), ^1^H NMR, and ^13^C NMR spectra. TLC was performed on aluminum-backed silica gel plates (Merck DC, Alufolien Kieselgel 60 F254) with spots visualized by UV light. All solvents were reagent grade and, when necessary, were purified and dried by standard methods. The concentration of solutions after reactions and extractions involved the use of a rotary evaporator operating at a reduced pressure of ca. 20 Torr. Organic solutions were dried over anhydrous sodium sulfate. Elemental analysis has been used to determine the purity of the described compounds that is > 95%. Analytical results are within ± 0.40% of the theoretical values. All chemicals were purchased from Sigma-Aldrich, Milan (Italy) or Alfa Aesar, Karlsruhe (Germany) and were of the highest purity.

#### Benzyl (4-(4-(quinolin-4-ylamino) benzamido) phenyl)carbamate (MC3353)

Triethylamine (0.37 mmol, 0.05 mL) and benzyl chloroformate (0.28 mmol, 0.04 mL) were slowly added to a cooled (0 °C) solution of *N*-(4-aminophenyl)-4-(quinolin-4-ylamino)benzamide 1 [20] (0.1 g, 0.28 mmol) in tetrahydrofuran (5 mL), and the resulting mixture was stirred at room temperature for 1.5 h. Then, the reaction was quenched by water and extracted by dichloromethane (3 × 10 mL), washed with brine (3 × 10 mL), then dried with anhydrous sodium sulphate, filtered, and concentrated under reduced pressure. The oily residue was purified by column chromatography (SiO_2_ eluting with ethyl acetate/methanol 10/1) to provide pure MC3353. Melting point: 220–222 °C; recrystallization solvent: acetonitrile/methanol; yield: 72%; ^1^H NMR (DMSO-d_6_, 400 MHz, *δ*; ppm) *δ* 5.16 (s, 2H, -CH2Ph), 7.20–7.49 (m, 10H, benzene protons), 7.58–7.60 (t, 1H, quinoline proton), 7.67–7.76 (m, 3H, benzene protons and quinoline proton), 7.92–8.01 (m, 3H, benzene protons), 8.37–8.39 (d, 1H, quinoline proton), 8.57–8.59 (d, 1H, quinoline proton), 9.24 (bs, 1H, -NH), 9.72 (bs, 1H, -NHCOOBn), 10.09 (bs, 1H, -NHCOPh) ppm; ^13^C NMR (DMSO-d6, 100 MHz, *δ*; ppm) *δ* 66.8, 111.4 (2C), 112.8, 121.6, 121.8 (4C), 124.2 (2C), 125.7, 127.1 (2C), 127.6, 128.9 (2C), 129.2, 129.6, 130.2 (2C), 133.5, 133.6, 136.1, 138.7, 149.3, 149.7, 151.6, 153.8, 164.7 ppm; MS (EI), m/z [M]^+^ C_30_H_24_N_4_O_3_ calculated 488.1848, found 488.1852. Elemental analysis: calculated %: C, 73.76; H 4.95; N 11.47. Found %: C, 73.88; H, 5.06; N, 11.20.

#### Dissolution of compounds

5-AZA (Sigma-Aldrich, Milan, Italy) was solubilized in a HOAc:H_2_O (1:1) solution at 200 mM. All other compounds including RG108 (synthetized as previously described in [17]), SGI-1027 (synthetized as previously described in [20]), DAC (Sigma-Aldrich, Milan, Italy), and MC3353 were resuspended in DMSO (Sigma-Aldrich, Milan, Italy) at 100 mM, 50 mM, 10 mM, and 1 mM, respectively.

### DNA methyltransferase assays

#### DNMT1 assay

His-DNMT1 (182 kDa, human) was cloned, expressed, and purified as described by Lee et al. [15]. The DNMT1 assay was performed according to Gros et al. [25]. Briefly, the DNMT1 enzymatic assay is based on the use of radiolabeled SAM, and the methylation occurs in homogeneous phase in 384-well microplates. The reaction is performed with DNMT1 at the final concentration of 90 nM in a total volume of 10 μL including also the chemical compound to be tested at the desired concentration, 1.25 μM of SAM//[methyl-^3^H] SAM (3TBq/mmol) mix in a ratio of 3:1 and 0.3 μM of biotinylated DNA duplex. DNMT1, as “maintenance” methyltransferase, requires hemimethylated DNA for the reaction. After 2 h incubation at 37 °C, 8 μL of the reaction’s solution is transferred into a streptavidin 96-well scintillant coated Flashplate^TM^ containing 190 μL of 20 mM SAH in 50 mM Tris-HCl (pH 7.4). The Flashplate^TM^ is then agitated at room temperature for 1 h and washed. The plate is read with TopCount (PerkinElmer, Villebon-sur-Yvette, France). The greater the DNMT1 inhibition, the lower will be the scintillation signal correlated to the incorporation of the tritiated methyl groups. In this assay, the negative and positive controls were defined as wells without enzyme and wells without any compound but just DMSO, respectively.

#### DNMT3A assay

DNMT3A enzyme inhibition was adapted from the restriction-based fluorescence assay protocol described in Ceccaldi et al. [18]. Briefly, it is performed in a 384-well microplate in a total volume of 50 μL. An oligonucleotide labeled at the 5′ end with biotin is hybridized to his complementary strand labeled at the 3′ end with 6-carboxyfluorescein. The DNA duplex thus formed contains only one single CpG site overlapping with a restriction site of a methylation sensitive restriction enzyme. The duplex is transferred in the wells coated with avidin. Thus, the duplex is fixed to the plate. The methylation reaction is performed by adding human C-terminal DNMT3A at the final concentration of 4 ng/μL in a total volume of 50 μL in the presence of the chemical compound to be tested at the desired concentration and the cofactor SAM at the final concentration of 20 μM. After incubation (1 h at 37 °C), the plate is washed and the methylation sensitive restriction enzyme HpyCH4IV is added. After 1-h incubation at 37 °C, the plate is washed and the fluorescence measured with a PerkinElmer Envision Multilabel Plate Reader (PerkinElmer, Villebon-sur-Yvette, France). The data are expressed as a percentage of inhibition vs. log concentration (*M*). Data are normalized referring to the “restriction control” (wells coated with labeled duplex not treated nor exposed to DNMT3A, but only cleaved by HpyCH4IV) as the maximum of inhibition (100%) and to the “DMSO control” (wells coated with labeled duplex but treated just with 0.1% DMSO exposed to DNMT3A and then to HpyCH4IV) as the minimum of inhibition (0%; total methylation).

#### CMV-luc assay in KG-1 cells

The assay was carried out as described by Rilova et al. [26]. The KG-1 cell line was stably transfected with the luciferase firefly (Luc^+^ from pGL3 by Promega, Madison, WI, USA) reporter gene under the control of a methylated CMV promoter (from pEGFP-N1 by Clontech) and selected for the maintenance of the methylation of CMV and luciferase expression silencing (KG-1 CMV-luc). The stably integrated KG-1 CMV-luc cells are cultivated in RPMI-1640 medium (Lonza, Strasbourg, France), supplemented with 10% FCS (Lonza, Strasbourg, France) and 0.5 mg/mL of geneticin (Sigma-Aldrich, Saint-Quentin Fallavier, France), under 5% CO_2_, at 37 °C and seeded at 20,000 cells per well in 96-well plates. After 5 or 24 h of incubation in the presence of compounds or the solvent DMSO, the induction of the promoter is measured by quantification of luciferase with the Brite-lite assay system (PerkinElmer, Villebon-sur-Yvette, France) according to the manufacturer’s protocol. The luminescence is measured on an EnVision™ Multilabel Plate Reader (PerkinElmer, Villebon-sur-Yvette, France), and the data are expressed as fold induction compared to DMSO control. The mean of 2–4 experiments and its standard error are reported.

To determine the antiproliferative activity of MC3353 in KG-1 cells, the cells were seeded at day 0 in a 96-well plate and treated with test compound solutions at a dose range spanning from 3.2 × 10^−9^ to 1 × 10^−5^ M at days 1, 2, and 3. On day 4, cell viability was assessed using the ATPLite kit (PerkinElmer, Villebon-sur-Yvette, France).

#### UCHL1 promoter demethylation assay (luciferase activity induction) in HCT116 colon cancer cells

##### Cell culture and treatments

Human colon cancer HCT116 (ATCC, VA, USA) cells were propagated in McCoy’s 5A medium (Euroclone, Milan, Italy) with 10% fetal bovine serum (FBS; Euroclone), 2 mM l-glutamine (Euroclone), and antibiotics (100 U/mL penicillin, 100 g/mL streptomycin) (Euroclone). For the treatments with MC3353, 5-AZA, and DAC as positive controls, 2 × 10^4^ cells were seeded in 6-well plates in triplicate. Cells were treated every 2 days, and the medium was changed. On day 5, cells were observed under a fluorescent microscope. The nuclei were counterstained with DAPI (10 μg/mL, Invitrogen, Tournai, Belgium).

##### Fluorescence detection

Fluorescence of EGFP and DAPI in living cells was analyzed by Cytation™ 5 Cell Imaging Multi-Mode Reader (BioTeK, Milan, Italy).

##### Transfection

Before the transfection, the pUMLIEP vector was linearized by with *Cla*I restriction enzyme (New England Biolabs, Milan, Italy) and then it was methylated by *Sss*I-methylase (New England Biolabs, Milan, Italy) for 60 min. The methylated vector was transfected into HCT116 cells using Lipofectamine™2000 (Invitrogen, Monza, Italy) and Opti-MEM® (Gibco, Monza, Italy), and stably introduced clones were selected with puromycin (1.0 μg/mL, Sigma-Aldrich, Milan, Italy). The detailed protocol for the generation of cells highly responsive to DNA demethylating agents is described in [27].

### Cell lines and culture conditions

U-937 (acute myeloid leukemia), RAJI (Burkitt’s lymphoma), PC-3 (prostate cancer), and MDA-MB-231 (breast cancer) cell lines were obtained from the Deutsche Sammlung für Mikroorganismen and Zellkulturen (DSZM, Braunschweig, Germany) and were cultured in RPMI 1640 (Lonza, Verviers, Belgium) complemented with 10% FBS (Lonza, Verviers, Belgium) and 1% antibiotic-antimycotic (Lonza). RPMI1788 (normal B lymphocytes) and KG-1 (acute myeloid leukemia) cell lines were obtained from the American Type Culture Collection (ATCC) and were maintained in RPMI 1640 complemented with 20% FBS and IMDM complemented with 10% FBS, respectively. Cell lines were treated with compounds at the indicated concentrations in the exponential growth phase.

Peripheral blood mononuclear cells (PBMCs) from healthy donors were isolated as previously described [28]. Freshly isolated PBMCs were either cultured [28] and treated at 1.10^6^ cells/mL (non-proliferating PBMCs) or stimulated with phytohaemoagglutinin (PHA) and interleukin (IL)-2 (called PHA_PBMCs) to induce blastogenesis in T lymphocytes before treatments as previously described [29]. Briefly, freshly isolated PBMCs were seeded at a concentration of 2.10^6^ cells/mL in RMPI 1640 supplemented with 1% antibiotic-antimycotic (Lonza, Verviers, Belgium) and 10% human AB serum (Corning, Fisher scientific, Merelbeke, Belgium). After overnight incubation, floating lymphoid cells were collected and seeded at 1.10^6^ cells/mL in RMPI 1640 supplemented with 1% antibiotic-antimycotic, 5% FBS, 10% human AB serum, 1 μg/mL PHA (Gentaur, Kampenhout, Belgium), 50 U/mL IL-2 (Roche, Prophac, Luxembourg City, Luxembourg), 1% non-essential amino acids (Invitrogen, Luxembourg), 1% HEPES (Invitrogen, Luxembourg), 1% sodium pyruvate (Invitrogen, Luxembourg), and 0.1% β-mercaptoethanol (Invitrogen, Luxembourg). After 72 h, the cells were seeded at 1.10^6^ cells/mL in the same mitogenic growth medium described just above and treated with test compounds.

The patient-derived human OS cell lines Saos-2, U-2OS, and MG-63 were obtained from ATCC (1992). The patient-derived OS cell lines IOR/OS9, IOR/OS20, and IOR/SARG were established and previously characterized at the Laboratory of Experimental Oncology of Rizzoli Orthopedic Institute [30]. Cell lines were profiled for DNA copy number changes [31] and DNA methylation status at approximately 27,000 CpG sites [32]*.* The cell line PDX-OS#1-C4 was obtained from OS patient-derived xenograft (PDX) after 4 passages in the animal. All cell lines were tested for mycoplasma contamination (Mycoalert Mycoplasma Detection Kit, Lonza, Verviers, Belgium) before use and authenticated by short-tandem repeat polymerase chain reaction (STR PCR) analysis (last control December 2017) using PowerPlex ESX fast System kit (Promega, Madison, WI, USA). The following loci were verified: AMEL, D3S1358, TH01, D21S11, D18S51, D10S1248, D1S1656, D2S1338, D16S539, D22S1054, VWA, D8S1179, FGA, D2S441, D12S391, D19S433, and SE33. All cell lines were immediately amplified to constitute liquid nitrogen stocks and were never passaged for more than 1 month upon thawing. Cells were maintained in Iscove’s modified Dulbecco’s medium (IMDM) supplemented with 10% heat-inactivated FBS (Lonza, Verviers, Belgium), penicillin (20 U/mL), and streptomycin (100 μg/mL) (Sigma-Aldrich, Milan, Italy) in 37 °C humidified at 5% CO_2_.

### Cell proliferation experiments

DKO HCT116 (ATCC, VA, USA) cells were propagated in Dulbecco’s modified Eagle medium (DMEM) (Euroclone, Milan, Italy) with 10% fetal bovine serum (FBS; Euroclone, Milan, Italy), 2 mM l-glutamine (Euroclone, Milan, Italy) and antibiotics (100 U/mL penicillin, 100 g/mL streptomycin) (Euroclone, Milan, Italy).

The MTT [3-(4,5-dimethylthiazol-2-yl)-2,5-diphenyltetrazolium bromide] (Sigma-Aldrich, Milan, Italy) assay was used to determine the proliferation of HCT116 clone and DKO HCT116 cells after treatment with MC3353, 5-AZA, and DAC. 5 × 104 cells/well for both cell lines were plated in 24-well plate and treated, in duplicate, with compounds at several concentrations for 24 and 48 h of induction. After treatments, MTT solution was added for 3 h at 0.5 mg/mL, the purple formazan crystals were dissolved in DMSO (Sigma-Aldrich, Milan, Italy), and the absorbance was read at a wavelength of 570 nm with reader TECAN M-200.

Proliferation and viability of U-937, KG-1, RAJI, PC-3, MDA-MB-231, RPMI1788, PBMCs, and PHA_PBMCs were assessed by trypan blue exclusion assays at the indicated time points. Nuclear morphology changes associated with apoptosis and necrosis were evaluated by fluorescence microscopy after Hoechst and propidium iodide (PI) staining as previously described [33].

To perform osteosarcoma cell growth inhibition experiments, 2 × 10^5^ cells/well were plated, and MC3353 (0.1–30 μM) or DAC (0.5 μM) was added after 24 h. Cells were exposed for up to 96 h before being counted by Trypan blue vital cell count (Sigma-Aldrich, Milan, Italy). In parallel, the cells were treated with DMSO-containing medium as a control. The highest final concentration of DMSO in the medium was < 0.005%, and DMSO had no effect on cell growth.

### RNA extraction, reverse transcription, and quantitative PCR in PC-3 and HCT116 cells for EMT pathway evaluation

RNAs were extracted by ReliaPrep™ RNA Tissue Miniprep (Promega, Madison, WI, USA) and reverse transcribed with PrimeScript RT Master Mix (Takara, Kusatsu, Shiga, Japan). cDNAs were amplified by a qPCR reaction using GoTaq qPCR Master Mix (Promega) and analyzed with the oligonucleotide pairs specific for the target genes. Relative amounts, determined with the 2^(−ΔCt)^ method, were normalized with respect to the human housekeeping gene L32. The primers used are as follows: L32 (forward: 5′-GGAGCGACTGCTACGGAAG-3′, reverse: 5′-GATACTGTCCAAAAGGCTGGAA-3′), E-cadherin (forward: 5′-TACGCCTGGGACTCCACCTA-3′, reverse: 5′-CCAGAAACGGAGGCCTGAT-3′), and MMP2 (forward: 5′-ATGCCGCCTTTAACTGGAG-3′, reverse: 5′-GGAAAGCCAGGATCCATTTT-3′).

### Western blots

Cells were lysed in Laemmli buffer; subsequently, the proteins were resolved by sodium dodecyl sulfate-polyacrylamide gel electrophoresis and transferred to a 0.45-μm nitrocellulose membrane (Bio-Rad Laboratories, Hercules, CA). The following primary antibodies were used for immunoblotting: α-E-cadherin (BD Transduction Laboratories, Franklin Lakes, NJ), α-MMP2 (Abcam), α-DNMT3a (Santa Cruz Biotechnologies, Dallas, TX), and α-GAPDH (Millipore Corp., Bedford, MA), used as a loading control. The immune complexes were detected with horseradish peroxidase-conjugated species-specific secondary antiserum (Bio-Rad Laboratories, Milan, Italy) then by enhanced chemiluminescence reaction (Bio-Rad Laboratories, Milan, Italy). Densitometric analysis of protein expression was performed by using the Fiji ImageJ image processing package.

### Osteosarcoma (Saos-2) differentiation towards osteoblasts

Four days after seeding, Saos-2 cells were exposed to specific osteogenic medium (IMDM supplemented with 2% FBS, 5 mM β-glycerophosphate, and 50 μg/mL ascorbic acid, Sigma-Aldrich, Milan, Italy) without (control) or with MC3353 (0.5–1 μM) or DAC (0.1–0.5 μM) and maintained in differentiating conditions for up to 21 days. The medium was renewed every 4 days, and the cells were harvested at various time points to collect total RNA and verify bone mineralization. Specific osteoblastic markers were evaluated by quantitative real-time PCR (RT-qPCR) as previously described [34]. The primers or assays (Applied Biosystems, Foster City, CA, USA) used are as follows: GAPDH (forward: 5′-GAAGGTGAAGGTCGGAGTC-3′, reverse: 5′-GAAGATGGTGATGGGATTTC-3′ and probe: 5′-CAAGCTTCCCGTTCTCAGCC-3′), OCN (forward: 5′-GGGCTCCCAGCCATTGAT-3′, reverse: 5′-CAAAGCCTTTGTGTCCAAGCA-3′), ALP (Hs01029144_m1), and COL1A2 (Hs01028970_m1). Amplification reactions were performed using a ViiA7™ Real-Time PCR System (Life Technologies, Carlsbad, CA, USA). To visualize bone mineralization (osteoblastic differentiation) after 7–21 days of MC3353 or DAC treatment, the plates were stained with 40 mM Alizarin red stain (ARS, Sigma-Aldrich, Milan, Italy). ARS staining was visualized with an ECLIPSE 90i microscope (Nikon, Minato, Tokyo, Japan) equipped with Plan fluor 10× NA 0.3. Images were acquired with an autocapture setting using a digital color camera (Nikon DS5 MC) and NIS-Elements AR3.10 software (Nikon, Minato, Tokyo, Japan).

### IC_50_ values determination and statistical analyses

IC_50_ values of compounds against cellular viability were determined by using nonlinear regression fitting curves with GraphPad Prism 8 or CalcuSyn software The *t* test used for statistical analyses was performed with GraphPad Prism 8. All the tests were one-tailed, and a *p* value < 0.05 was considered statistically significant.

## Results

### Synthesis of MC3353

**Scheme 1:**
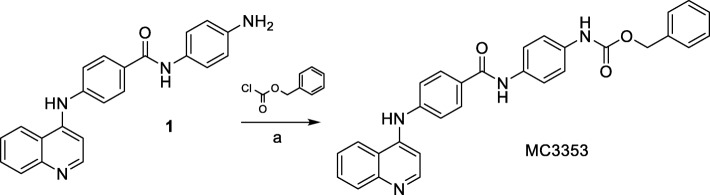
Synthesis of MC3353. Reagents and conditions: (a): (C_2_H_5_)_3_N, anhydrous THF, 0 °C, 1.5 h

MC3353 was prepared by treating the known *N*-(4-aminophenyl)-4-(quinolin-4-ylamino)benzamide 1 [20], dissolved in dry tetrahydrofuran, with benzyl chloroformate in the presence of triethylamine (Scheme 1).

### DNA methylation inhibiting assays

MC3353 was tested against human DNMT1 (hDNMT1) and the C-terminal catalytic domain of human DNMT3A (hDNMT3A) to assess its inhibitory activity. RG-108 and SGI-1027 were used as reference drugs (Table 1).

**Table 1:** EC_50_ inhibition data of MC3353 against the enzymatic activity of human DNMT1 and human DNMT3A through radioactivity and fluorescent assay, respectively [18, 25]

Compound	EC_50_ ± SD, μM	
	hDNMT1	hDNMT3A
MC3353	67 ± 4	17 ± 3
RG-108	390 ± 50	> 500
SGI-1027	12 ± 2	0.8 ± 0.2

EC_50_ are reported as the median concentration, at which a compound inhibits 50% of the DNA methylation and the standard deviation (SD) of at least two independent experiments for DNMT1 and DNMT3A

In enzyme assays, MC3353 showed increased potency compared to RG-108 and lower efficacy with respect to SGI-1027. In particular, it was 6-fold more potent than RG-108 and 5.6-fold less effective than SGI-1027 against hDNMT1, whereas against hDNMT3A, the potency displayed by MC3353 was > 29-fold higher than RG-108 and 20-fold lower than SGI-1027.

Afterwards, MC3353 was tested in KG-1 leukemia cells for its ability to reactivate gene expression in a stably integrated luciferase reporter construction under the control of a CMV promoter inhibited by DNA methylation (CMV-luc assay) [23, 26]. After 5 or 24 h of incubation in the presence of drugs at the indicated concentrations, the luciferase signal was measured and normalized to the value of the non-treated cells (Table 2). Since the CMV-luc assay is highly compromised when the tested compounds induce cell death, the cytotoxicity of MC3353 against KG-1 cells at 5 and 24 h was determined in parallel. MC3353 was highly toxic for KG-1 cells after 24 h of treatment starting from a concentration of 1 μM (Additional file 1: Table S1 and indicated in stars in Table 2).

**Table 2:** Fold induction of the luciferase signal of the CMV-luc construct in MC3353 or SGI-1027 treated KG-1 cells

Compound	Luciferase expression (fold ± SD^a^)					
	0.1 μM		1 μM		5 μM	
	5 h	24 h	5 h	24 h	5 h	24 h
MC3353	1.0 ± 0.05	1.1 ± 0.1	5.8 ± 0.9	4.4 ± 3.5*	6.7 ± 0.8*	ND
SGI-1027	1.2 ± 0.3	0.9 ± 0.1	1.0 ± 0.1	1.5 ± 0.1	4.0 ± 1.2	9.8 ± 4.7*

*ND* not detected, *SD* standard deviation

*Partial toxic effect on KG-1 cells induced strong variations on promoter induction

^a^All experiments were performed from *n* = 2 to *n* = 4

Nevertheless, 1 μM MC3353 strongly enhanced luciferase expression after 5 h of treatment, while the inductions observed at 1 μM after 24 h and at 5 μM after 5 h are partially impaired by MC3353-induced cytotoxicity (Table 2). Of note, MC3353 induced luciferase expression at a lower concentration compared to the reference drug SGI-1027.

**Fig. 2:**
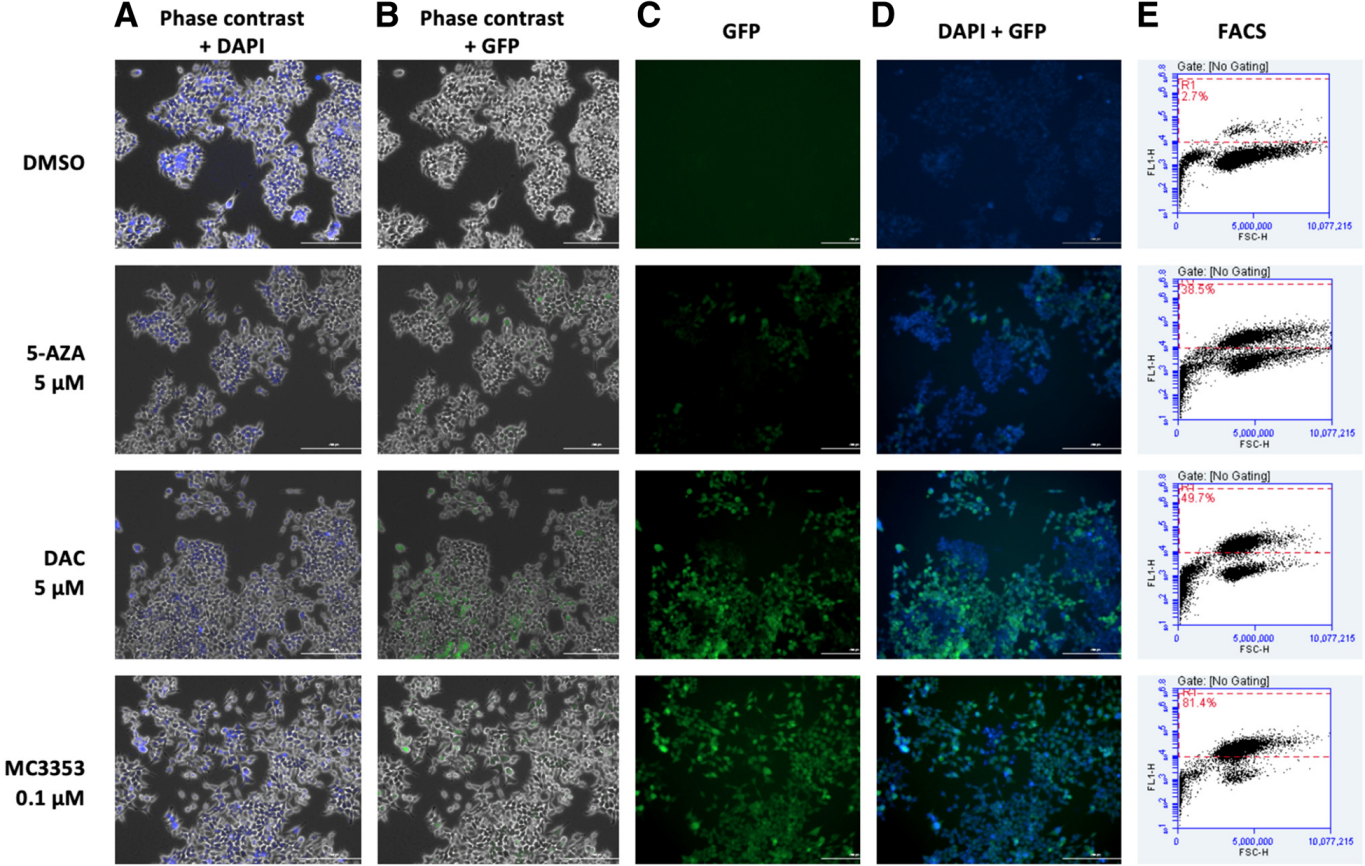
EGFP expression. MC3353 strongly demethylates pUCHL1 in human HCT116 colon cancer cells. Microscopy imaging (**a**–**d**) and FACS evaluation (**e**) of HCT116 cells transfected with a methylated pUCHL1 vector and treated for 5 days with DMSO as vehicle control (Ctr), 5-AZA, DAC, and MC3353 at indicated concentrations. The drugs were added every 2 days, and the medium was changed

Next, the demethylating activity of MC3353 was evaluated in cells transfected with luciferase and EGFP reporter genes driven by the UCHL1 promoter [27]. The UCHL1 promoter is silenced by methylation in human colon cancer [35] and can be demethylated by specific treatments, inducing expression of Metridia luciferase and EGFP reporter genes. In particular, the pUCHL1 vector, after methylation, was transfected into HCT116 colon cancer cells, and the stable clone was obtained by puromycin selection. Afterwards, cells were treated with 5-AZA (5 μM), with DAC (5 μM), or with MC3353 (0.1 μM) for 5 days. Such a low MC3353 dose was used because of its cytotoxicity at higher concentrations in long treatments. Microscopy and FACS analyses revealed that in comparison with control cells (DMSO), MC3353 strongly induced (81.4%) EGFP gene expression, witnessing a specific DNA demethylating activity in cells. Interestingly, MC3353 was markedly more effective than both 5-AZA and DAC, which displayed an induction of 38.5% and 49.7%, respectively, at a 50-fold higher concentration (Fig. 2).

**Fig. 3:**
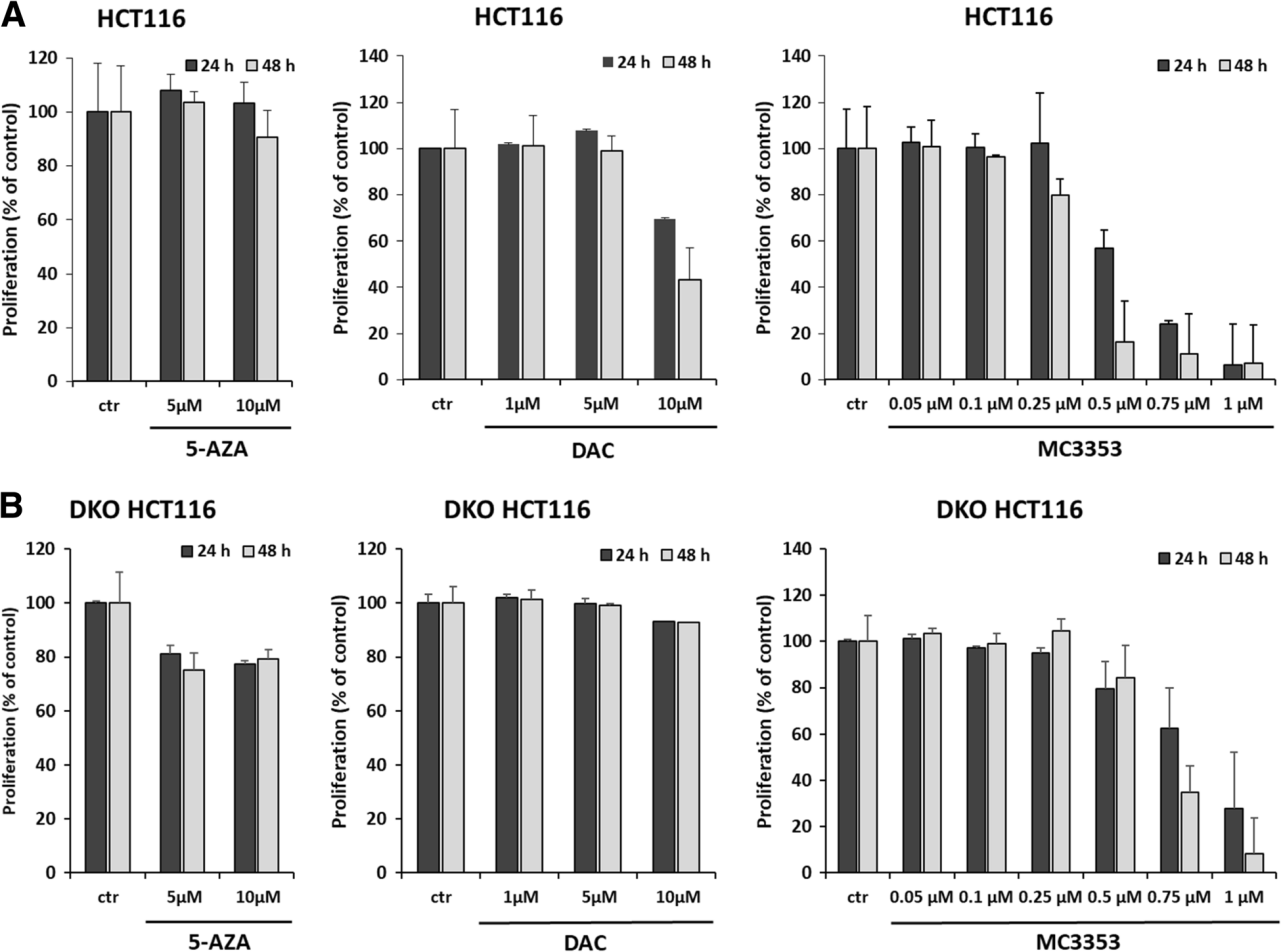
Effect of MC3353 on human HCT116 (**a**) and on human DKO HCT116 (**b**) colon cancer cell proliferation. Cells were treated with increasing doses of MC3353, 5-AZA, or DAC. After 24 and 48 h, cell proliferation was evaluated through MTT assay. Data represent the mean (± SD) of three independent experiments

Next, we sought to evaluate whether, along with its strong demethylating activity, MC3353 could impair cancer cell proliferation and whether this effect was DNMT-dependent. Accordingly, we tested the effect of increasing concentrations of MC3353 on the proliferation of wild type as well as double DNMT knockout (DKO) HCT116 colon carcinoma cells treated for 24 and 48 h [36]. Moreover, we compared the effect of MC3353 to the reference DNMTi 5-AZA and DAC. The results clearly demonstrate that MC3353 markedly decreased HCT116 proliferation starting at 0.5 μM after 48 h of incubation, whereas a negligible effect was observed after 5-AZA treatment. A modest proliferation inhibition was elicitated by DAC treatment, used at a 20-fold higher concentration (10 μM) than MC3353 (Fig. 3a). Interestingly, when tested against DKO HCT116 cells, MC3353 was markedly less efficient to impair cell proliferation at 0.5 μM after 48 h of treatment; nevertheless, at higher concentrations, increased cytotoxicity was observed. No evidence of cytotoxicity was shown by DAC treatment even at the highest dose, whereas a weak effect was triggered by 5-AZA (Fig. 3b).

### Effect of MC3353 on a panel of different cancer and healthy cell models

**Fig. 4:**
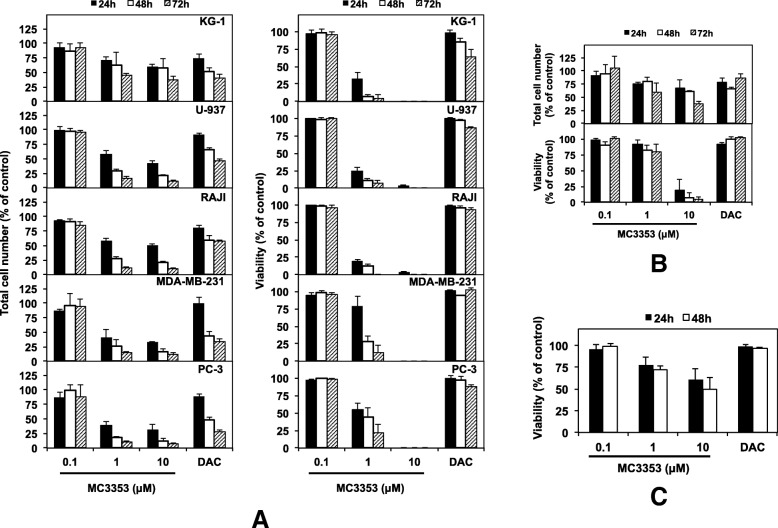
Effect of MC3353 on cell proliferation and viability. Cells were treated with increasing doses of MC3353 or 1 μM DAC. After 24, 48, and 72 h, cell proliferation and viability were evaluated using trypan blue exclusion staining. Data represent the mean (± SD) of three independent experiments. **a** Cancer and **b** normal peripheral blood B lymphocyte (RPMI1788) cell line and **c** PBMCs from healthy donors

To further extend our findings, MC3353 was also tested in a panel of cancer cell lines (KG-1 and U-937 acute myeloid leukemia, MDA-MB-231breast cancer, RAJI Burkitt’s lymphoma, and PC-3 prostate cancer) to assess its effects on cell proliferation and viability. The effect of MC3353 on proliferating normal B lymphocyte cell line RPMI1788 as well as non-proliferating and proliferating PBMCs from healthy donors was also determined to assess the differential toxicity. DAC (1 μM) has been used as a reference drug in these assays. MC3353 showed a greater potency than SGI-1027 against all tested cancer cell lines as well as healthy cell models. Nonetheless, due to the overall more potent effect on cancer cell viability compared to normal cell models, MC3353 displayed a better differential toxicity compared to the reference drug SGI-1027 (Fig. 4 and Table 3).

**Table 3:** Cell viability IC_50_ values for KG-1, U-937, RAJI, PC-3, MDA-MB-231, RPMI1788, and non-proliferating or proliferating PBM cells after 48 h of treatment with MC3353 or SGI-1027

Compound	IC_50_ ± SD^a^, μM (Selectivity index^b^)							
	KG-1	U-937	RAJI	PC-3	MDA-MB-231	RPMI1788	PBMCs	PHA_PBMCs^c^
MC3353	0.3 ± 0.0	0.4 ± 0.0	0.4 ± 0.0	0.9 ± 0.4	0.9 ± 0.7	2.4 ± 0.2	8.9 ± 4.2	4.6 ± 0.5
(IC_50_ RPMI1788/IC_50_ cell line)	(7.2)	(6.6)	(6.3)	(2.7)	(2.6)	(NA)	(NA)	(NA)
(IC_50_ PBMCs/IC_50_ cell line)	(26.2)	(24.0)	(23.1)	(9.8)	(9.7)	(NA)	(NA)	(NA)
(IC_50_ PHA_PBMCs/IC_50_ cell line)	(13.5)	(12.4)	(11.9)	(5.1)	(5.0)	(NA)	(NA)	(NA)
SGI-1027	3.6 ± 0.6	1.7 ± 1.5	7.8 ± 0.7	5.0 ± 0.6	8.5 ± 7.2	6.7 ± 1.3	18.1 ± 6.8	ND
(IC_50_ RPMI1788/IC_50_ cell line)	(1.9)	(3.9)	(0.9)	(1.3)	(0.8)	(NA)	(NA)	(NA)
(IC_50_ PBMCs/IC_50_ cell line)	(5)	(10.6)	(2.3)	(3.6)	(2.1)	(NA)	(NA)	(NA)

*SD* standard deviation, *NA* not applicable, *ND* not determined

^a^Data are the mean of at least three independent experiments

^b^Differential toxicity was calculated as the ratio of the IC_50_ normal cells (either PBMCs or RMPI1788) to the IC_50_ neoplastic cells

^c^PHA_PBMCs: phytohemagglutinin-stimulated PBMCs

**Fig. 5:**
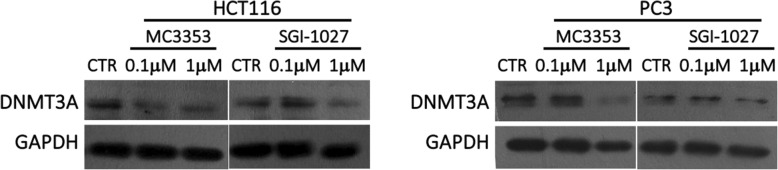
Western blot analysis of DNMT3A protein expression levels in HCT116 and PC-3 cells exposed for 24 h to the indicated concentrations of MC3353 or SGI-1027. GAPDH was used as a loading control. Blots are representative of two independent experiments

Due to the discrepancy between its high potency in cellular assays (at low micromolar/submicromolar level for the reactivation of genes silenced by promoter DNA methylation and the effects on cell viability in a panel of cancer cell lines) and its biochemical DNMT inhibition (micromolar range), MC3353 was screened against a panel of 46 kinases (ExpresS Diversity Kinase Profile, Cerep), taking into account its structural similarity (presence of aminoquinoline, *p*-aminobenzoic, and benzene-1,4-diamine fragments) with kinase inhibitors. However, 10 μM MC3353 induced an inhibition greater than 50% only against RAF1, SRC Proto-Oncogene, and Non-Receptor Tyrosine Kinase (Src) and tropomyosin receptor kinase A (TRKA) kinase activities (60, 57, and 67%, respectively), being much less potent or inactive against all the other tested enzymes (Additional file 1: Table S2). These results could, at least partly, explain the potent cytotoxic activity exerted by MC3353. Moreover, prompted by the evidence that SGI-1027 induced a selective downregulation of DNMT1 with minimal or no effects on DNMT3A in different cancer cell lines [19], and that the DNMTi MC3343 decreased the expression of all DNMTs in primary osteosarcoma cells [22], we tested MC3353 and the reference drug SGI-1027, in HCT116 and PC-3 cells to evaluate the putative MC3353 ability to provide a similar effect. We performed this assay only for DNMT3A, as it was the isoform more sensible to the enzymatic inhibition by MC3353. Hence, western blotting reveals that DNMT3A protein downregulation was observed in both cell lines (Fig. 5) and already detectable in HCT116 cells at the same concentration (0.1 μM) at which MC3353 displayed strong demethylation activity (Fig. 2). A higher dose (1 μM) was requested for achieving this result in PC-3 cells. A moderate effect on DNMT3A expression level was detectable upon treatment with SGI-1027 in mainly HCT116.

**Fig. 6:**
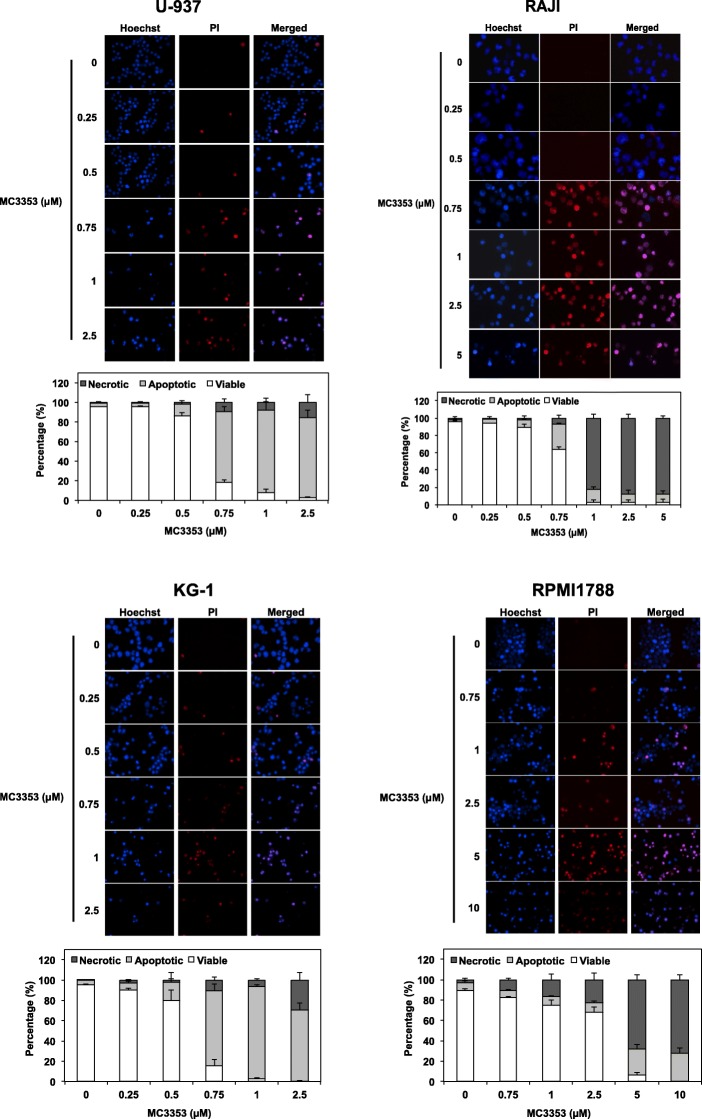
Nuclear morphology analysis in U-937, RAJI, KG-1, and RPMI1788 cells. Cells were treated with increasing doses of MC3353. Upper panels—after 72 h of treatment, the nuclear morphology was analyzed by fluorescence microscopy after Hoechst and PI staining. Pictures are representative of three independent experiments. Lower panels—the results of cell counting are represented as the mean (± SD) of three independent experiments

Next, we evaluated the cell death modality (i.e., apoptosis vs. necrosis) triggered by treatments with MC3353 in KG-1, U-937, and RAJI cancer cells as well as normal RPMI1788 cells by analyzing the nuclear morphological changes under fluorescence microscopy following Hoechst and PI staining (Fig. 6). In KG-1 and U-937 AML cells, MC3353 induced mainly apoptosis with a considerable increase between 0.5 and 1 μM. Similarly, in RAJI cells, there was a drastic increase in cell death rate between 0.5 and 1 μM, associated with a shift from apoptosis to necrosis (Fig. 6). Finally, in MC3353-treated RPMI1788 cells, we observed a mixed type of cell death induction, mainly necrosis, starting from a concentration of 5 μM.

### MC3353 reversed EMT by affecting E-cadherin and MMP2 expression

The EMT process is recognized as an important step of tumorigenesis. In ovarian cancer, it was shown that the majority of gene-specific transforming growth factor (TGF)-β-induced methylation changes occur in CpG islands located in or near promoters. Pathway analysis of the hypermethylated loci identified functional networks strongly associated with EMT and cancer progression, including cellular movement, cell cycle, organ morphology, cellular development, and cell death and survival. Altered methylation and corresponding expression of specific genes during TGF-β-induced EMT include E-cadherin and collagen 1A1. Moreover, TGF-β induces DNMT1, DNMT3A, and DNMT3B expression and activities, and treatment with the DNMTi SGI-110 prevents TGF-β-induced EMT [37, 38].

**Fig. 7:**
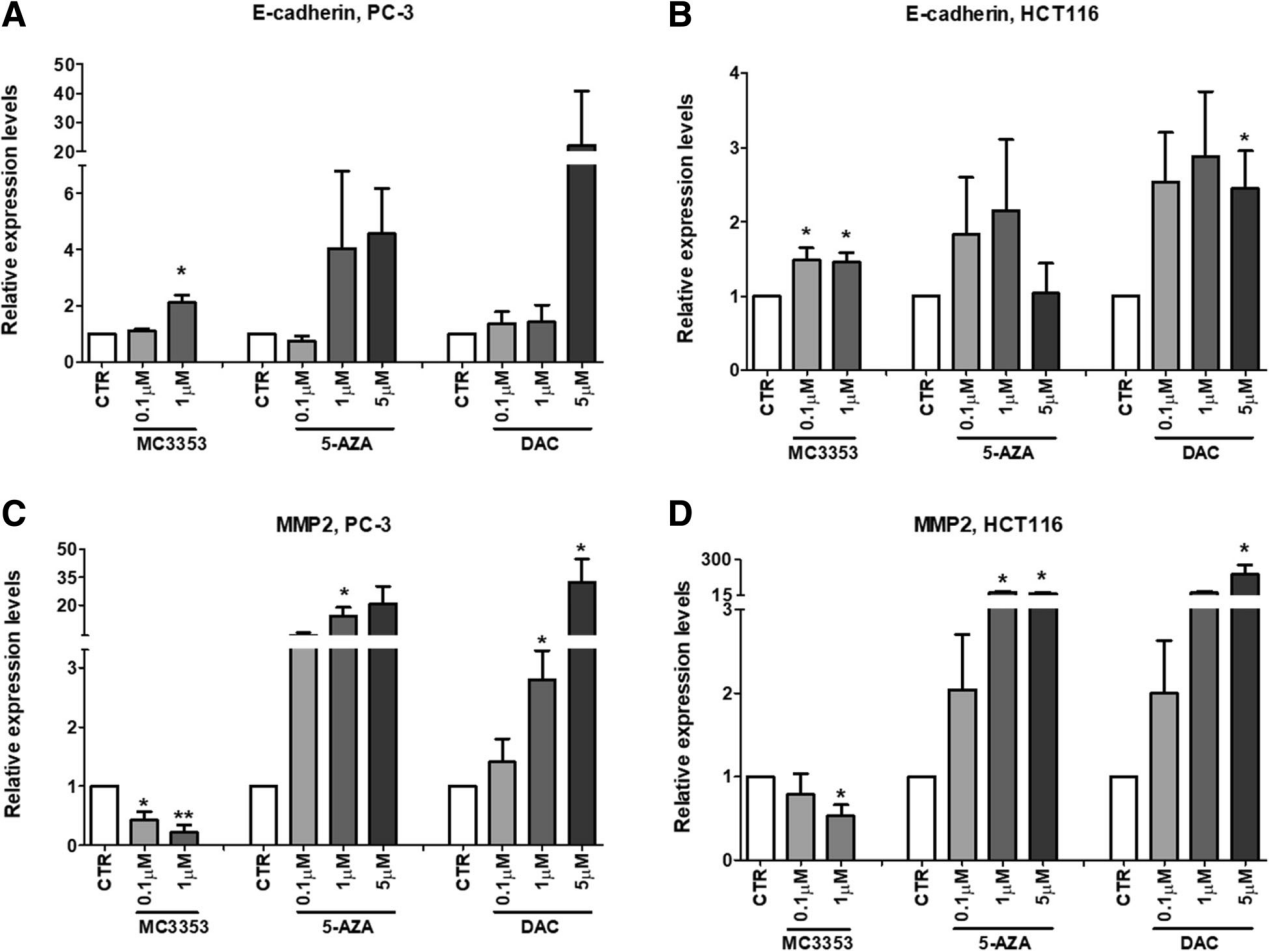
Effects of MC3353 on E-cadherin and MMP2 mRNA expression levels in PC-3 and HCT116 cancer cells. Cells were treated with vehicle (DMSO or AcOH) used as control (CTR), MC3353 for 24 h, or 5-AZA or DAC for 5 days. **a**, **b** E-cadherin and **c**, **d** MMP2 transcripts quantification via qPCR in PC-3 and HCT116 cancer cells. Each histogram represents the mean of four independent experiments. Significance is represented as *p < 0.05 and as **p < 0.01 related to the control groups

Prompted by these findings, we investigated the ability of MC3353 to modulate EMT pathway, using 5-AZA and DAC as reference drugs. Hence, MC3353 was tested in human PC-3 and HCT116 cells at 0.1 and 1 μM for 24 h (Fig. 7) while the treatment with 5-AZA and DAC was performed every 2 days for 5 days at 0.1, 1, and 5 μM. The levels of mRNA transcripts of E-cadherin and matrix metalloproteinase-2 (MMP2) were determined. E-cadherin is involved in the maintenance of the epithelial identity and in the control of tumor invasiveness, whereas MMP2, through degradation of type IV collagen, the most abundant component of the basement membrane, controls an essential step for metastatic progression of most cancers as well as for induction of angiogenesis [39].

The results clearly show that treatment with MC3353 induced E-cadherin mRNA expression transcripts at a very low and non-cytotoxic concentration (0.1 μM) in HCT116 colon cancer cells, whereas in PC-3 cells, this effect was observed only at 1 μM dose. On the other hand, opposite results were found analyzing MMP2 expression, which was strongly downregulated especially in PC-3 cells. Notably, while 5-AZA and DAC are able to induce E-cadherin expression in both cell lines (at higher doses in PC-3 cells, from 1 to 5 μM), they are unable to downregulate MMP2 expression, indeed they increased MMP2 level in both cell lines also at lower doses (0.1 to 1 μM).

**Fig. 8:**
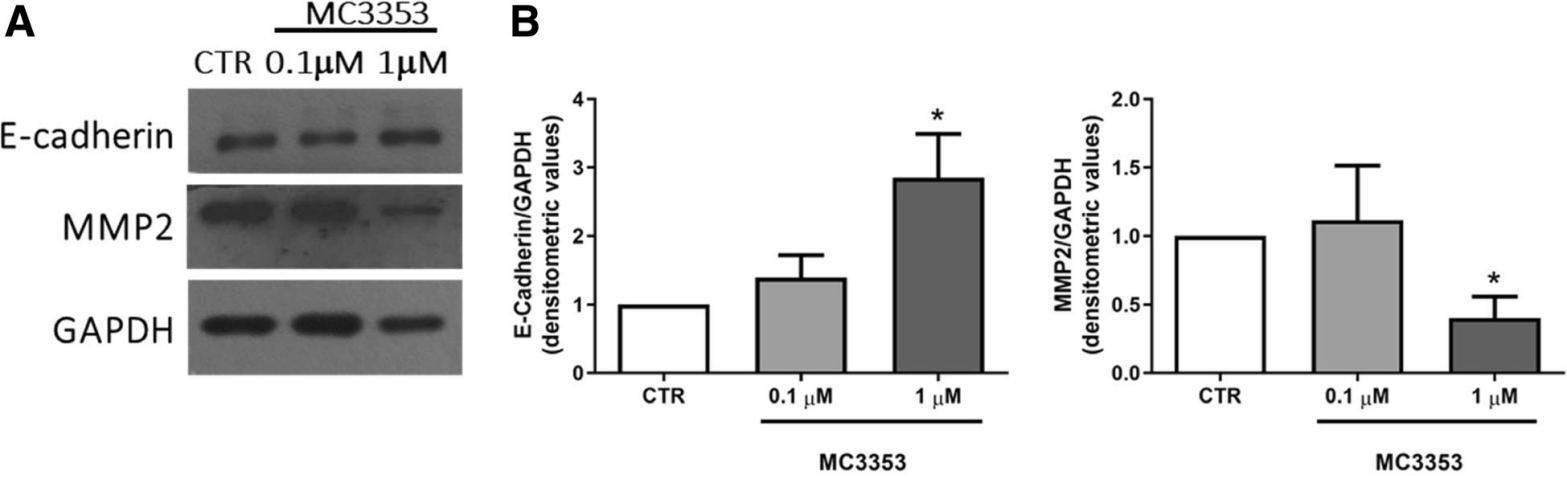
Effects of MC3353 on E-cadherin and MMP2 protein expression levels in PC-3 cancer cells. Cells were treated with vehicle (DMSO) used as control (CTR) or MC3353 for 24 h. **a** E-cadherin and MMP2 protein detection by western blot. GAPDH was used as a loading control. Blots are representative of three independent experiments. **b** Densitometric analysis of E-cadherin and MMP2 protein expression. Significance is represented as *p < 0.05 related to the control groups. Data were obtained from three independent experiments and expressed as mean ± SEM

In order to verify at the protein levels E-cadherin and MMP2 regulation after MC3353 treatment, we analyzed the protein expression in the PC-3 cells, in which the changes are more relevant (Fig. 8).

The western blotting and its relative densitometric analysis highlight that the treatment with MC3353 significantly induced E-cadherin expression while reduced MMP2 protein levels at 1 μM dose.

### MC3353 impairs proliferation and modulates genes expression and osteoblast differentiation in osteosarcoma cells

**Fig. 9:**
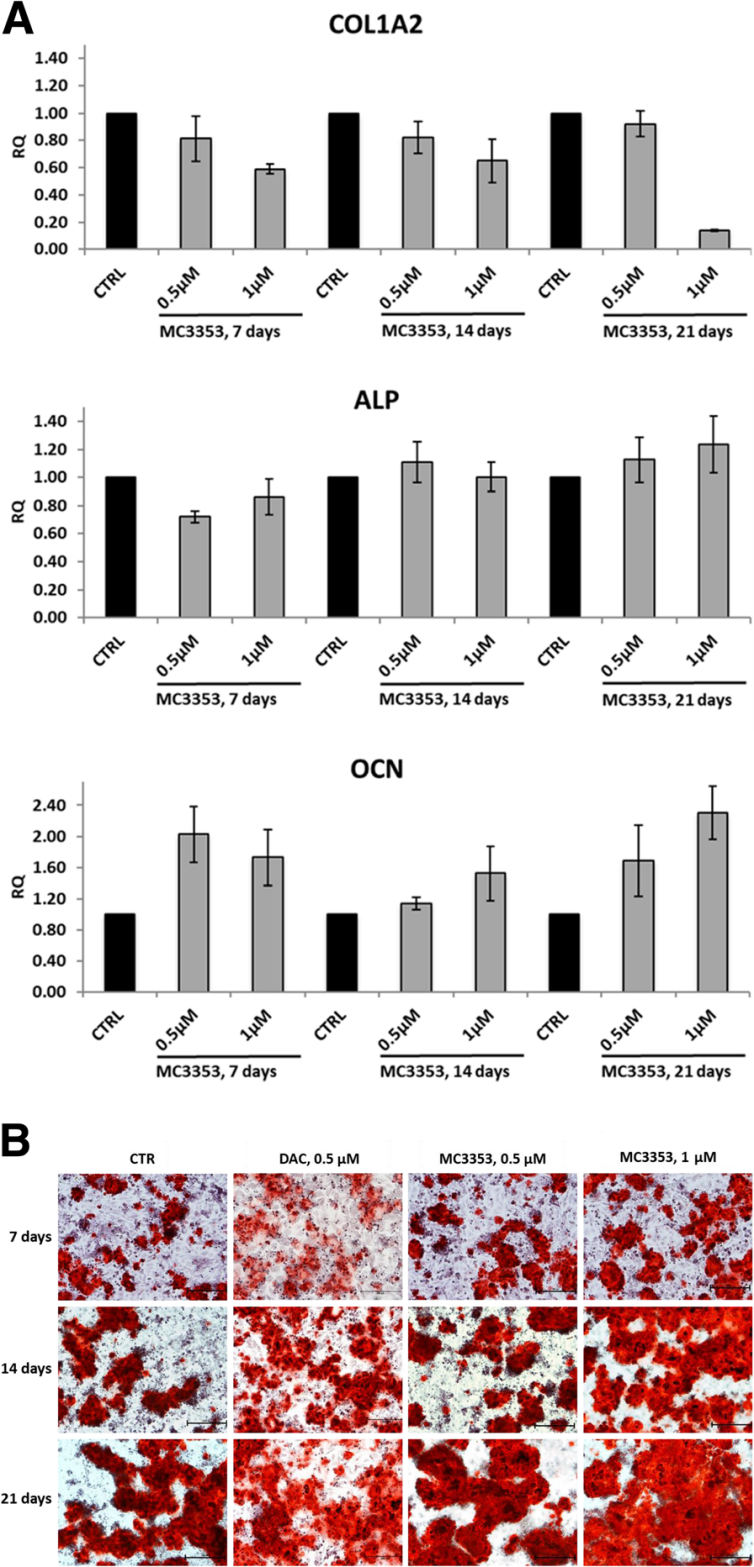
Effects of MC3353 on osteoblastic differentiation in osteosarcoma cells. **a** qRT-PCR expression of representative osteoblast differentiation markers in Saos-2 osteosarcoma cells treated with MC3353 at indicated concentrations under osteoblastic conditions. COL1A2, ALP, and OCN are displayed to sign early, intermediate, and late stages of the differentiation process, respectively. The relative mRNA expression levels of target genes (RQ) were calibrated on untreated cells (CTR) at each time point. Data are the mean of at least two independent experiments; SD, standard deviation. **b** ARS staining of a mineralized matrix over 7–21 days of Saos-2 cells treatment with MC3353 or DAC at indicated concentrations in differentiation medium. Scale bar indicates 200 μm

The promising results observed in vitro and in vivo with MC3343 treatments of osteosarcoma cells and mouse model [22] encouraged us to investigate if its analog MC3353 could prove anticancer properties in osteosarcoma as well. Hence, the antiproliferative potential of MC3353 was tested against patient-derived Saos-2, U-2OS, MG63, IOR/OS9, IOR/OS20, IOR/SARG, and PDX-OS#1-C4 cells. The results clearly highlight that, similarly to the strong arrest of cell proliferation displayed in HCT116, KG-1, U-937, RAJI, PC-3, and MDA-MB-231, was able to impair the osteosarcoma cells growth with low single-digit micromolar IC_50_, being comparable (Saos-2 and U-2OS) or more efficient (IOR/OS9, IOR/OS20 and IOR/SARG cells) than DAC, used as reference drug. Only in two osteosarcoma cell lines, namely MG63 and PDX-OS#1-C4 MC3353, were approximately tenfold or threefold less potent than DAC (Table 4). Next, we investigated the effects of the DNMTi on the differentiation of Saos-2 cell line, which displays osteoblast-like features and the ability to differentiate toward the osteogenic lineage when maintained in low-serum medium containing ascorbic acid (50 μg/mL) and β-glycerophosphate (5 mM) [34]. Osteoblasts produce and secrete proteins that constitute the bone matrix, such as collagens and osteocalcin, and are essential for osteoid matrix mineralization, a process mediated by alkaline phosphatase. Accordingly, we evaluated the effects of MC3353 on the expression of genes involved in the osteoblast differentiation such as COL1A2, ALP, and OCN by RT-PCR (Fig. 9a), as well as on the production of a mineralized matrix by ARS staining (Fig. 9b). Thus, when tested at 0.5 and 1 μM and along 3 weeks, MC3353 dose-dependently decreased COL1A2 expression, an early marker of osteoblast differentiation, whereas it increased OCN expression, a marker of terminal osteoblast differentiation. The overall efficacy of MC3353 in the modulation of the three genes was more evident at day 21. Only at the most extended exposure, some weak induction of ALP expression was detected. Moreover, MC3353 proved to augment mineralized matrix production, even better than 5-AZA, and this effect was nicely observable after ARS staining (Fig. 9b).

**Table 4:** IC_50_ values of cellular viability for osteosarcoma cell lines (Saos-2, U-2OS, MG63, IOR/OS9, IOR/OS20, IOR/SARG, PDX-OS#1-C4) after 96 h of treatment with MC3353 or DAC

Compound	IC_50_ ± SDa, μM						
	Saos-2	U-2OS	MG63	IOR/OS9	IOR/OS20	IOR/SARG	PDX-OS#1-C4
MC3353	1.1 ± 0.3	1.4 ± 0.1	2.4 ± 1.7	2.0 ± 0.1	1.5 ± 0.1	1.5 ± 0.1	2.0 ± 0.1
DAC	1.0 ± 0.9	1.1 ± 0.9	0.2 ± 0.2	5.7 ± 3.4	9.2 ± 2.2	5.4 ± 1.0	0.7 ± 0.6

*SD* standard deviation

^a^Data are the mean of at least two independent experiments

## Discussion

Aberrant DNA methylation of gene promoter regions is presently recognized to be highly involved in human malignancies. Two nucleosides DNMTi, 5-AZA, and DAC have been approved by the US FDA to treat myelodysplasia; however, since they suffer from low selectivity and water instability, combined to metabolic liability, the discovery and identification of non-nucleoside DNMTi and their validation in cancer are highly pursued.

In 2014, we described the discovery of MC3343 as a novel hit DNMTi showing higher potency and selectivity than SGI-1027 with respect to other AdoMet-dependent enzymes and a different mechanism of action. In cancer cells, MC3343 showed similar antiproliferative potency but less toxicity than SGI-1027. In medulloblastoma cancer stem cells, MC3343 displayed arrest of proliferation and cytodifferentiation induction, and in osteosarcoma cells, it affected tumor proliferation in vitro and in vivo and provided osteoblastic differentiation by targeting and re-expressing specific genes.

Based on these findings, we designed a novel analog of SGI-1027, MC3353, by replacing the 4-methyl-2, 6-diaminopyrimidine moiety with a benzyl carbamate group. When tested against human DNMTs, MC3353 displayed micromolar inhibition of DNMT1 and DNMT3A in enzymatic assays. To validate its ability to demethylate DNA in cells, MC3353 was tested in leukemia KG-1 cells, where it was able to induce activation of the luciferase gene under the control of a methylated promoter in a CMV-luc reporter assay at 1 μM. In parallel to this expression induction effect in leukemia cells, in HCT116 cells, MC3353 was able to strongly induce EGFP gene expression (81.4%), which is under the UCHL1 promoter, silenced by methylation in colon cancer, hence underlining its specific demethylating activity at a very low concentration (0.1 μM). In this assay, MC3353 was markedly more effective than both 5-AZA and DAC, which induced EGFP gene expression at 5 μM of 38.5% and 49.7%, respectively, thus being the approved drugs less potent at a 50-fold higher concentration.

Moreover, to correlate its demethylating activity to antiproliferative effect, MC3353 was further tested in HCT116 where it proved an efficient antiproliferative agent already at 0.5 μM, being a lot stronger than either 5-AZA or DAC. Noteworthy, when tested against DKO HCT116 cells, MC3353 evidently displayed less potent antiproliferative effect at 0.5 μM, so proving that the strong proliferation arrest shown at this dose in HCT116 cells was, at least in part, DNMT-dependent. However, augmented cytotoxicity was highlighted in DKO HCT116 cells upon treatment with MC3353 at higher doses, thus suggesting some off-target engagement by MC3353. Next, our DNMT inhibitor was screened against a panel of different cancer cell lines (KG-1, U-937, RAJI, PC-3, and MDA-MB-231 cells), where it was more potent than SGI-1027 and DAC in impairing cell viability (IC_50_ values in the low micromolar/submicromolar range). Although MC3353 was also more toxic against normal proliferating and non-proliferating PBMCs and RPMI1788 cells, due to its high potency, MC3353 displayed improved selectivity against cancer cell lines compared to SGI-1027 in the tested models. The type of cell death induced by MC3353 was mainly apoptosis in KG-1 and U-937 cells and necrosis in RAJI and RPMI1788 lines. In U-937 cells, MC3353 initially induced apoptosis and then necrosis at higher concentrations. Since MC3353 acts as a DNMTi at concentrations over 10 μM in the biochemical assay and its potency in cancer cells is rather at low single-digit micromolar or submicromolar level, we decided to investigate the additional mechanism(s) to explain this uncorrelation. To address this issue, a screening against 46 kinases was performed, and MC3353 was found to inhibit over 50% RAF1, Src, and TRKA kinase activities at a fixed dose of 10 μM. This additional target engagement may contribute in part to the high cellular potency highlighted by MC3353. Nevertheless, as already observed in different cancer cell lines after SGI-1027 and MC3343 treatment, also the novel MC3353 provided protein degradation of DNMT3A, the enzyme mainly inhibited. This effect was revealed in both PC-3 and HCT116 cells, the latter being the cell line where MC3353 displayed strong demethylation activity and at the same dosage. This result also well correlates with the potent effect observed in all the cell proliferation assays; indeed, DNMT3A degradation could give a contribution to the MC3353 efficacy in all cellular experiments here studied. In addition, in PC-3 and HCT116 cells, this compound reversed the EMT process by inducing E-cadherin (mainly in HCT116) and reducing MMP2 (mainly in PC-3) mRNA transcripts levels at low and non-cytotoxic concentrations (0.1 μM) and after 24 h of treatment. With respect to MMP2, we found that both 5-AZA and DAC, able to markedly induce E-cadherin expression particularly at higher doses, result unable to downregulate its expression, in accordance with previous studies [40–42]. Interestingly, both SGI-1027 and MC3353 were found to induce the downregulation of DNMT3A protein expression, indicating their role not only on DNMT activity but also on protein levels.

Next, MC3353 was also tested against several primary osteosarcoma cell lines, where previously, its analog MC3343 displayed inhibition of tumor proliferation and induction of osteoblastic differentiation through the specific re-expression of related genes. MC3353 induced COL1A2 downregulation together with ALP and mainly OCN upregulation after 3 weeks of treatment mainly at 1 μM. Moreover, this compound increased also mineralized matrix production after 3 weeks of treatment, as evidence of the effect on differentiation from osteosarcoma to osteoblast cells, even more efficiently than DAC, used as a reference.

## Conclusions

In conclusion, the present study reveals that the novel DNMTi MC3353 displayed a stronger demethylating activity than both 5-AZA and DAC in cell models through both enzyme inhibition and protein downregulation, and it was able to re-activate a methylation-silenced gene such as UCHL1 in HCT116. Its dose- and time-dependent antiproliferative activity was accompanied by induction of cell death, differentiation, and genes modulation, but also by impairment of the EMT process, as shown by its E-cadherin and MMP2 modulating effect. To the best of our knowledge, MC3353 is the first non-nucleoside DNMTi with an effect on EMT pathway, opening a novel scenario for biological investigation on migration/invasiveness.

## Additional file

Additional file 1: **Table S1** and ** Table S2**. Supporting information. (DOCX 94 kb)

## Abbreviations

5-AZA: 5-Azacytidine
ALP: Alkaline phosphatase
ARS: Alizarin red stain
AdoMet: *S*-adenosyl-l-methionine
CMV: Cytomegalovirus
CMV-luc: Cellular luciferase CMV reporter system
COL1A2: Collagen 1
DAC: 5-Aza-2′-deoxycytidine
DAPI: 4′,6-Diamidin-2-phenylindol
DKO: Double knockout
DMSO: Dimethylsulfoxide
DNA: Deoxyribonucleic acid
DNMT: DNA methyltransferase
DNMTi: DNMT inhibitor
EGFP: Enhanced green fluorescent protein
EIMS: Electron ionization mass spectrometry
EMT: Epithelial-mesenchymal transition
FACS: Fluorescence-activated cell sorting
FBS: Fetal bovine serum
IL: Interleukin
MMP2: Matrix metalloproteinase 2
NMR: Nuclear magnetic resonance
OCN: Osteocalcin
PBMC: Peripheral blood mononuclear cell
PDX: Patient-derived xenograft
PHA: Phytohemagglutinin
PI: Propidium iodide
qPCR: Quantitative polymerase chain reaction
RNA: Ribonucleic acid
SEM: Standard error of the mean
Src: Sarcoma
STR PCR: Short-tandem repeat polymerase chain reaction
TGF-β: Transforming growth factor-β
TLC: Thin layer chromatography
TRKA: Tropomyosin receptor kinase A
TSG: Tumor suppressor gene
UCHL1: Ubiquitin C-terminal hydrolase L1
US FDA: United States Federal Drug Administration
