# Correction: Identification of a novel quinoline-based DNA demethylating compound highly potent in cancer cells

**DOI:** 10.1186/s13148-022-01339-y

**Published:** 2022-09-28

**Authors:** Clemens Zwergel, Michael Schnekenburger, Federica Sarno, Cecilia Battistelli, Maria Cristina Manara, Giulia Stazi, Roberta Mazzone, Rossella Fioravanti, Christina Gros, Frédéric Ausseil, Cristina Florean, Angela Nebbioso, Raffaele Strippoli, Toshikazu Ushijima, Katia Scotlandi, Marco Tripodi, Paola B. Arimondo, Lucia Altucci, Marc Diederich, Antonello Mai, Sergio Valente

**Affiliations:** 1 Department of Chemistry and Technologies of Drugs, Sapienza University of Rome, P.le A. Moro 5, 00185 Rome, Italy; 2 Laboratoire de Biologie Moléculaire et Cellulaire du Cancer, Hôpital Kirchberg, 9 rue Edward Steichen, L-2540 Luxembourg City, Luxembourg; 3 Department of Medicine of Precision, University of Studi della Campania Luigi Vanvitelli, Vico L. De Crecchio 7, 80138 Naples, Italy; 4 Department of Molecular Medicine, Sapienza University of Rome, Viale Regina Elena 324, 00161 Rome, Italy; 5 Laboratory of Experimental Oncology, IRCCS - Istituto Ortopedico Rizzoli, via di Barbiano, 1/10, 40136 Bologna, Italy; 6 Center for High-Throughput Chemical Biology, Simon Fraser University, 8888 University Drive, Burnaby, BC V5A 1S6 Canada; 7 Pierre Fabre Laboratories, 3 Avenue Hubert Curien, 31100 Toulouse, France; 8 National Institute for Infectious Diseases L. Spallanzani, IRRCCS, Via Portuense, 292, 00149 Rome, Italy; 9 Division of Epigenomics, National Cancer Center Research Institute, 5-1-1 Tsukiji, Chuo-ku, Tokyo, 104-0045 Japan; 10 Pasteur Institute, Cenci-Bolognetti Foundation, Sapienza University of Rome, P.le A. Moro 5, 00185 Rome, Italy; 11 Epigenetic Chemical Biology, Institut Pasteur, CNRS UMR3523, 28 rue du Docteur Roux, 75724 Paris, France; 12 Department of Pharmacy, Research Institute of Pharmaceutical Sciences, College of Pharmacy, Seoul National University, 1 Gwanak-ro, Gwanak-gu, 08826 Korea

## Correction: Clinical Epigenetics (2019) 11:68 10.1186/s13148-019-0663-8

Figure 6 of the original publication [1] contained a picture duplication in the U-937 panel resulting from an error while copy/pasting the individual pictures used to prepare the figure. The erroneous picture panel did not affect the corresponding quantification of cell death and the interpretation of the results as it was done with the correct picture set. The corrected Fig. 6 is presented in this correction.

**Fig. 6:**
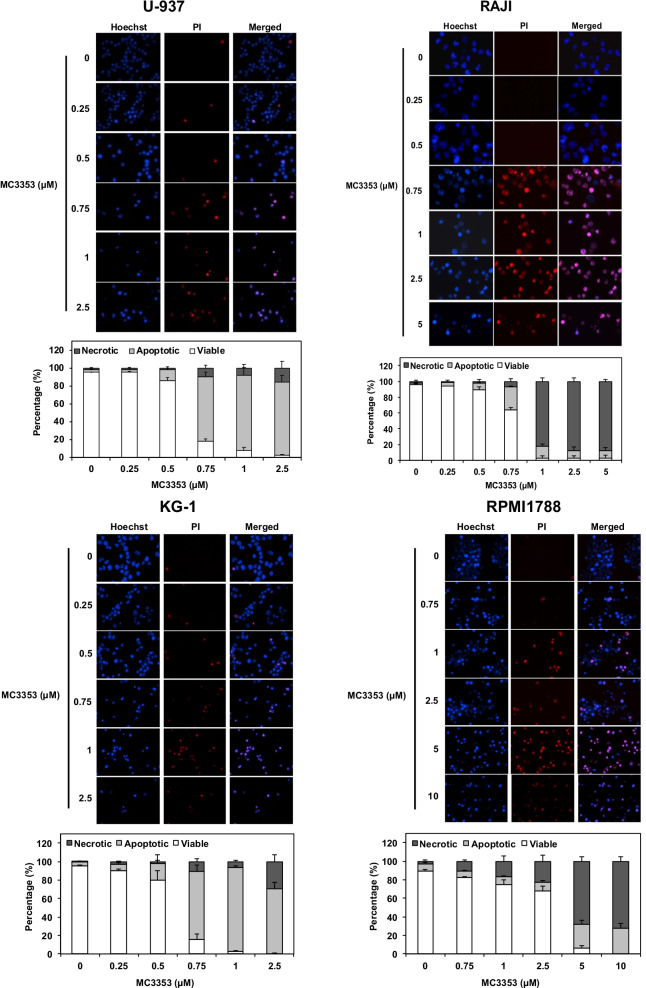
Nuclear morphology analysis in U-937, RAJI, KG-1, and RPMI1788 cells. Cells were treated with increasing doses of MC3353. Upper panels—after 72 h of treatment, the nuclear morphology was analyzed by fluorescence microscopy after Hoechst and PI staining. Pictures are representative of three independent experiments. Lower panels—results of cell counting are represented as the mean (± SD) of three independent experiments
