# Supplementary material for: Identification of a novel quinoline-based DNA demethylating compound highly potent in cancer cells
Source: Clin Epigenetics. 2019 May 6;11:68. doi: 10.1186/s13148-019-0663-8 (PMC6501426; doi:10.1186/s13148-019-0663-8)
Supplement: Supplementary file 1 — Table S1 and Table S2. Supporting information. (DOCX 94 kb) [file 13148_2019_663_MOESM1_ESM.docx]

Supplementary Material

**Identification of a Novel Quinoline-Based DNA Demethylating Compound Highly Potent in Cancer Cells**

**Clemens Zwergel, Michael Schnekenburger, Federica Sarno, Cecilia Battistelli, Maria Cristina Manara, Giulia Stazi, Roberta Mazzone, Rossella Fioravanti, Christina Gros, Frédéric Ausseil, Cristina Florean, Angela Nebbioso, Raffaele Strippoli, Toshikazu Ushijima, Katia Scotlandi, Marco Tripodi, Paola B. Arimondo, Lucia Altucci, Marc Diederich, Antonello Mai* and Sergio Valente*******

*** Correspondence:** [Antonello.mai@uniroma1.it](mailto:Antonello.mai@uniroma1.it), [Sergio.valente@uniroma1.it](mailto:Sergio.valente@uniroma1.it)

# Supplementary Data

**Table S1.** Antiproliferative activity of different doses of MC3353 in KG-1 cells after 5 and 24 h treatment.

| **Compd** | **% inhibition of proliferation, KG-1 cells** | | | | | | | |
| --- | --- | --- | --- | --- | --- | --- | --- | --- |
|  | **0.1 µM** | | **1 µM** | | **5 µM** | | **10 µM** | |
|  | **5h** | **24h** | **5h** | **24h** | **5h** | **24h** | **5h** | **24h** |
| **MC3353** | 5.5 | 2.9 | 17.7 | 98.7 | 81.2 | 100 | 96.8 | 100 |

**Table S2.** Screening of MC3353 on a panel of kinases: Percentage of inhibition at 10 µM

| **kinases** | **% inhibition at 10 µM by MC3353** | **Enzyme quantity (ng)^a^** | **buffer^b^** | **substrate** | **[s]^c^** | **[ATP]** | **Incubation time** | **Antibody** | **Reference compound^d^** | **Ref. (lit)** |
| --- | --- | --- | --- | --- | --- | --- | --- | --- | --- | --- |
| Abl kinase (h) | 0 | 0.4 | A | Ulight-TK peptide | 100 nM | 10 μM | 30 min | anti-phospho-PT66 |  | [1] |
| Akt1/PKBalpha (h) | -261 | 1.2 | B | Ulight-CREBtide (CKRREILSRRPSYRK) | 25 nM | 30 μM | 60 min | anti-phospho-CREB |  | [2] |
| AurA/Aur2 kinase (h) | 4 | 2.36 | B | Ulight-RRRSLLE (PLK) | 100 nM | 10 μM | 15 min | anti-phospho-PLK |  | [3] |
| CaMK2alpha (h) | -22 | 8.1 | C | Ulight-CGSGSGRPRTSSFAEG (Crosstide) | 50 nM | 10 μM | 30 min | anti-phospho-Crosstide | AIP | [4] |
| CDC2/CDK1 (h) (cycB) | -25 | 3 | B | Ulight-CFFKNIVTPRTPPPSQGK-amide (MBP) | 100 nM | 10 μM | 15 min | anti-phospho-MBP |  | [5] |
| CHK1 (h) | 7 | 0.6 | B | Ulight-CREBtide (CKRREILSRRPSYRK) | 25 nM | 30 μM | 30 min | anti-phospho-CREB |  | [6] |
| CHK2 (h) | -30 | 1.32 | B | Ulight-CREBtide (CKRREILSRRPSYRK) | 25 nM | 30 μM | 15 min | anti-phospho-CREB |  | [7] |
| c-Met kinase (h) | -116 | 0.4 | B + 300 nM poly-D-Lys | Ulight-CAGAGAIETDKEYYTVKD (JAK1) | 25 nM | 10 μM | 60 min | anti-phospho-PT66 |  | [8] |
| EGFR kinase (h) | 5 | 0.0452 | A + 100 nM poly-D-Lys | Ulight-CAGAGAIETDKEYYTVKD (JAK1) | 100 nM | 10 μM | 15 min | anti-phospho-PT66 | PD153035 | [9] |
| EphA2 kinase (h) | -295 | 0.2 | A | Ulight-TK peptide | 50 nM | 10 μM | 30 min | anti-phospho-PT66 |  | [10] |
| EphA3 kinase (h) | -244 | 0.56 | A | Ulight-TK peptide | 50 nM | 30 μM | 60 min | anti-phospho-PT66 |  | [11] |
| EphB4 kinase (h) | -726 | 0.05 | B | Ulight-TK peptide | 100 nM | 50 μM | 90 min | anti-phospho-PT66 |  | [12] |
| ERK2 (h) (P42mapk) | -21 | 1.38 | B | Ulight-CFFKNIVTPRTPPPSQGK-amide (MBP) | 100 nM | 10 μM | 15 min | anti-phospho-MBP |  | [13] |
| FGFR1 kinase (h) | -200 | 0.252 | B | Ulight-CAGAGAIETDKEYYTVKD (JAK1) | 100 nM | 100 μM | 60 min | anti-phospho-PT66 |  | [14] |
| FGFR2 kinase (h) | 19 | 0.0075 | B + 50 nM poly-D-Lys | Ulight-CAGAGAIETDKEYYTVKD (JAK1) | 25 nM | 10 μM | 15 min | anti-phospho-PT66 |  | [15] |
| FGFR3 kinase (h) | -169 | 0.7 | A | Ulight-CAGAGAIETDKEYYTVKD (JAK1) | 100 nM | 10 μM | 90 min | anti-phospho-PT66 |  | [16] |
| GSK3beta (h) | -29 | 21.9 | B | Ulight-CFFKNIVTPRTPPPSQGK-amide (MBP) | 100 nM | 10 μM | 90 min | anti-phospho-MBP |  | [17] |
| HGK (h) (MAP4K4) | -358 | 19.5 | B | Ulight-FLGFTYVAP (P70S6K) | 50 nM | 1 μM | 90 min | anti-phospho-P70S6K |  | [18] |
| IKKalpha (h) | 18 | 11.2 | B | Ulight-IkappaB-alpha | 100 nM | 5 μM | 30 min | anti-phospho-IkappaB-alpha |  | [19] |
| IRAK4 (h) | -235 | 16.72 | B | Ulight-FLGFTYVAP (P70S6K) | 100 nM | 2.5 μM | 90 min | anti-phospho-P70S6K |  | [20] |
| IRK (h) (InsR) | 38 | 0.0156 | B | Ulight-Poly GAT[EAY(1:1:1)]n | 50 nM | 30 μM | 10 min | anti-phospho-PT66 |  | [21] |
| JAK3 (h) | -316 | 0.204 | B | Ulight-CAGAGAIETDKEYYTVKD (JAK1) | 100 nM | 0.5 μM | 60 min | anti-phospho-PT66 |  | [22] |
| JNK1 (h) | -32 | 6.8 | B | Ulight-CFFKNIVTPRTPPPSQGK-amide (MBP) | 100 nM | 10 μM | 60 min | anti-phospho-MBP |  | [23] |
| KDR kinase (h) (VEGFR2) | -111 | 0.88 | B | Ulight-CAGAGAIETDKEYYTVKD (JAK1) | 100 nM | 25 μM | 60 min | anti-phospho-PT66 |  | [24] |
| Lck kinase (h) | 4 | 1 | B | Ulight-Poly GAT[EAY(1:1:1)]n | 25 nM | 10 μM | 10 min | anti-phospho-PT66 |  | [1] |
| MAPKAPK2 (h) | -214 | 2.44 | B | Ulight-CREBtide (CKRREILSRRPSYRK) | 25 nM | 1 μM | 15 min | anti-phospho-CREB |  | [25] |
| MARK1 (h) | -116 | 11.6 | B | Ulight-RRRSLLE (PLK) | 50 nM | 1 μM | 30 min | anti-phospho-PLK |  | [26] |
| MNK2 (h) | 21 | 5 | B | Ulight-CREBtide (CKRREILSRRPSYRK) | 25 nM | 100 μM | 90 min | anti-phospho-CREB |  | [27] |
| MST4 kinase (h) | -243 | 5.2 | B | Ulight-CRFARKGSLRQKNV (PKC) | 50 nM | 10 μM | 30 min | anti-phophohistone H3 |  | [28] |
| NEK2 (h) | -9 | 2.728 | B | Ulight-FLGFTYVAP (P70S6K) | 50 nM | 10 μM | 60 min | anti-phospho-P70S6K |  | [29] |
| p38alpha kinase (h) | -58 | 6 | B | Ulight-CFFKNIVTPRTPPPSQGK-amide (MBP) | 100 nM | 100 μM | 30 min | anti-phospho-MBP | SB202190 | [30] |
| PAK2 (h) | -135 | 17.6 | B | Ulight-RRRSLLE (PLK) | 50 nM | 50 μM | 60 min | anti-phospho-PLK |  | [31] |
| PAK4 (h) | -4 | 20 | B | Ulight-RRRSLLE (PLK) | 50 nM | 1 μM | 30 min | anti-phospho-PLK |  | [32] |
| PDK1 (h) | -149 | 50 | B | Ulight-FLGFTYVAP (P70S6K) | 400 nM | 10 μM | 90 min | anti-phospho-P70S6K |  | [33] |
| Pim2 kinase (h) | 20 | 6.36 | B | Ulight-CREBtide (CKRREILSRRPSYRK) | 25 nM | 3 μM | 60 min | anti-phospho-CREB |  | [34] |
| PKA (h) | 27 | 0.005 | A | Ulight-PLK (Ser 137) | 50 nM | 1 μM | 10 min | anti-phospho-PLK |  | [35] |
| PKCbeta 2 (h) | 11 | 0.06 | B | Ulight-CREBtide (CKRREILSRRPSYRK) | 25 nM | 30 μM | 15 min | anti-phospho-CREB |  | [36] |
| PLK1 (h) | -263 | 9.6 | B | Ulight-FLGFTYVAP (P70S6K) | 40 nM | 5 μM | 60 min | anti-phospho-P70S6K |  | [37] |
| RAF-1 kinase (h) | 60 | 5 | B | Ulight-ARTKQTARKSTGGKAPRKQLAGCG (histone H3) | 50 nM | 10 μM | 180 min | anti-phospho-histone H3 |  | [38] |
| ROCK1 (h) | -51 | 8.2 | B | Ulight-RRRSLLE (PLK) | 50 nM | 1 μM | 30 min | anti-phospho-PLK |  | [39] |
| SGK1 (h) | 29 | 3.45 | A | Ulight-RRRSLLE (PLK) | 50 nM | 10 μM | 30 min | anti-phospho-PLK |  | [40] |
| SIK (h) | 33 | 9 | B | Ulight-CREBtide (CKRREILSRRPSYRK) | 25 nM | 30 μM | 90 min | anti-phospho-CREB | Ro-318220 | [41] |
| Src kinase (h) | 57 | 0.06 | B | Ulight-Poly GAT[EAY(1:1:1)]n | 5 nM | 5 μM | 10 min | anti-phospho-PT66 |  | [42] |
| TAOK2 (TAO1) (h) | -462 | 12.8 | B | Ulight-FLGFTYVAP (P70S6K) | 40 nM | 5 μM | 60 min | anti-phospho-P70S6K |  | [43] |
| TRKA (h) | 67 | 0.86 | B | Ulight-Poly GAT[EAY(1:1:1)]n | 5 nM | 100 μM | 10 min | anti-phospho-PT66 |  | [44] |

^a^Amount of enzyme (expressed in nanograms) in 10 μL reaction volume; ^b^ Buffer composition: A: 40 mM Hepes/ Tris (pH 7.4), 0.8 mM EGTA/Tris, 8 mM MgCl_2_, 3.6 mM DTT, 0.008% Tween 20; B: 40 mM Hepes/ Tris (pH 7.4), 0.8 mM EGTA/Tris, 8 mM MgCl_2_, 1.6 mM DTT, 0.008% Tween 20; C: 40 mM Hepes/Tris (pH 7.4), 0.8 mM EGTA/Tris, 8 mM MgCl_2_, 2.5 mM CaCl_2_, 1.6 mM DTT, 0.008% Tween 20, and 5 µg/ml calmodulin; ^c^ substrate concentration; ^d^ Only when different from staurosporine.

**Experimental procedure of kinase assays**

The test compound, reference compound or water (control) were mixed with the enzyme (for the exact amount see table below) in the appropriate buffer. Thereafter, the reaction was initiated by adding the required amount of the appropriate substrate and of ATP, and the mixture is incubated at room temperature (variable reaction time, see table below). For control basal measurements, the enzyme was omitted from the reaction mixture. Following incubation, the reaction was stopped by adding 13 mM EDTA. After 5 min, the proper antibody labelled with europium chelate was added. After 60 more min, the fluorescence transfer was measured at λ_ex_=337 nm, λ_em_=620 nm and λ_em_=665 nm using a microplate reader (Envision, Perkin Elmer). The enzyme activity was determined by dividing the signal measured at 665 nm by that measured at 620 nm (ratio). The results are expressed as a percent inhibition of the control enzyme activity. The standard inhibitory reference compound is staurosporine when not differently indicated (see table below), which has been tested in each experiment at several concentrations to obtain an inhibition curve from which its IC_50_ value is calculated.

General information

- Assay volume and format: 10 µL in 384-well plate
- Compound addition: [100x] solution in solvent then [5x] solution in water
- Maximum tolerable DMSO concentration: 1%

**References**

1. Park YW, Cummings RT, Wu L, Zheng S, Cameron PM, Woods A, Zaller DM, Marcy AI, Hermes JD: **Homogeneous proximity tyrosine kinase assays: scintillation proximity assay versus homogeneous time-resolved fluorescence.** *Anal Biochem* 1999, **269:**94-104.

2. Barnett SF, Defeo-Jones D, Fu S, Hancock PJ, Haskell KM, Jones RE, Kahana JA, Kral AM, Leander K, Lee LL, et al: **Identification and characterization of pleckstrin-homology-domain-dependent and isoenzyme-specific Akt inhibitors.** *Biochem J* 2005, **385:**399-408.

3. Sun C, Newbatt Y, Douglas L, Workman P, Aherne W, Linardopoulos S: **High-throughput screening assay for identification of small molecule inhibitors of Aurora2/STK15 kinase.** *J Biomol Screen* 2004, **9:**391-397.

4. Ishida A, Fujisawa H: **Stabilization of calmodulin-dependent protein kinase II through the autoinhibitory domain.** *J Biol Chem* 1995, **270:**2163-2170.

5. Meijer L, Borgne A, Mulner O, Chong JP, Blow JJ, Inagaki N, Inagaki M, Delcros JG, Moulinoux JP: **Biochemical and cellular effects of roscovitine, a potent and selective inhibitor of the cyclin-dependent kinases cdc2, cdk2 and cdk5.** *Eur J Biochem* 1997, **243:**527-536.

6. Zhao B, Bower MJ, McDevitt PJ, Zhao H, Davis ST, Johanson KO, Green SM, Concha NO, Zhou BB: **Structural basis for Chk1 inhibition by UCN-01.** *J Biol Chem* 2002, **277:**46609-46615.

7. Ng CP, Lee HC, Ho CW, Arooz T, Siu WY, Lau A, Poon RY: **Differential mode of regulation of the checkpoint kinases CHK1 and CHK2 by their regulatory domains.** *J Biol Chem* 2004, **279:**8808-8819.

8. Bardelli A, Longati P, Gramaglia D, Basilico C, Tamagnone L, Giordano S, Ballinari D, Michieli P, Comoglio PM: **Uncoupling signal transducers from oncogenic MET mutants abrogates cell transformation and inhibits invasive growth.** *Proc Natl Acad Sci U S A* 1998, **95:**14379-14383.

9. Weber W, Bertics PJ, Gill GN: **Immunoaffinity purification of the epidermal growth factor receptor. Stoichiometry of binding and kinetics of self-phosphorylation.** *J Biol Chem* 1984, **259:**14631-14636.

10. Kinch MS, Moore MB, Harpole DH, Jr.: **Predictive value of the EphA2 receptor tyrosine kinase in lung cancer recurrence and survival.** *Clin Cancer Res* 2003, **9:**613-618.

11. Sharfe N, Freywald A, Toro A, Roifman CM: **Ephrin-A1 induces c-Cbl phosphorylation and EphA receptor down-regulation in T cells.** *J Immunol* 2003, **170:**6024-6032.

12. Sturz A, Bader B, Thierauch KH, Glienke J: **EphB4 signaling is capable of mediating ephrinB2-induced inhibition of cell migration.** *Biochem Biophys Res Commun* 2004, **313:**80-88.

13. Bardwell AJ, Abdollahi M, Bardwell L: **Docking sites on mitogen-activated protein kinase (MAPK) kinases, MAPK phosphatases and the Elk-1 transcription factor compete for MAPK binding and are crucial for enzymic activity.** *Biochem J* 2003, **370:**1077-1085.

14. Mohammadi M, McMahon G, Sun L, Tang C, Hirth P, Yeh BK, Hubbard SR, Schlessinger J: **Structures of the tyrosine kinase domain of fibroblast growth factor receptor in complex with inhibitors.** *Science* 1997, **276:**955-960.

15. Robertson SC, Meyer AN, Hart KC, Galvin BD, Webster MK, Donoghue DJ: **Activating mutations in the extracellular domain of the fibroblast growth factor receptor 2 function by disruption of the disulfide bond in the third immunoglobulin-like domain.** *Proc Natl Acad Sci U S A* 1998, **95:**4567-4572.

16. Hart KC, Robertson SC, Kanemitsu MY, Meyer AN, Tynan JA, Donoghue DJ: **Transformation and Stat activation by derivatives of FGFR1, FGFR3, and FGFR4.** *Oncogene* 2000, **19:**3309-3320.

17. Meijer L, Skaltsounis AL, Magiatis P, Polychronopoulos P, Knockaert M, Leost M, Ryan XP, Vonica CA, Brivanlou A, Dajani R, et al: **GSK-3-selective inhibitors derived from Tyrian purple indirubins.** *Chem Biol* 2003, **10:**1255-1266.

18. Yao Z, Zhou G, Wang XS, Brown A, Diener K, Gan H, Tan TH: **A novel human STE20-related protein kinase, HGK, that specifically activates the c-Jun N-terminal kinase signaling pathway.** *J Biol Chem* 1999, **274:**2118-2125.

19. Huynh QK, Boddupalli H, Rouw SA, Koboldt CM, Hall T, Sommers C, Hauser SD, Pierce JL, Combs RG, Reitz BA, et al: **Characterization of the recombinant IKK1/IKK2 heterodimer. Mechanisms regulating kinase activity.** *J Biol Chem* 2000, **275:**25883-25891.

20. Li S, Strelow A, Fontana EJ, Wesche H: **IRAK-4: a novel member of the IRAK family with the properties of an IRAK-kinase.** *Proc Natl Acad Sci U S A* 2002, **99:**5567-5572.

21. al-Hasani H, Passlack W, Klein HW: **Phosphoryl exchange is involved in the mechanism of the insulin receptor kinase.** *FEBS Lett* 1994, **349:**17-22.

22. Zhou YJ, Hanson EP, Chen YQ, Magnuson K, Chen M, Swann PG, Wange RL, Changelian PS, O'Shea JJ: **Distinct tyrosine phosphorylation sites in JAK3 kinase domain positively and negatively regulate its enzymatic activity.** *Proc Natl Acad Sci U S A* 1997, **94:**13850-13855.

23. Bennett BL, Sasaki DT, Murray BW, O'Leary EC, Sakata ST, Xu W, Leisten JC, Motiwala A, Pierce S, Satoh Y, et al: **SP600125, an anthrapyrazolone inhibitor of Jun N-terminal kinase.** *Proc Natl Acad Sci U S A* 2001, **98:**13681-13686.

24. Itokawa T, Nokihara H, Nishioka Y, Sone S, Iwamoto Y, Yamada Y, Cherrington J, McMahon G, Shibuya M, Kuwano M, Ono M: **Antiangiogenic effect by SU5416 is partly attributable to inhibition of Flt-1 receptor signaling.** *Mol Cancer Ther* 2002, **1:**295-302.

25. Tan Y, Rouse J, Zhang A, Cariati S, Cohen P, Comb MJ: **FGF and stress regulate CREB and ATF-1 via a pathway involving p38 MAP kinase and MAPKAP kinase-2.** *EMBO J* 1996, **15:**4629-4642.

26. Timm T, Marx A, Panneerselvam S, Mandelkow E, Mandelkow EM: **Structure and regulation of MARK, a kinase involved in abnormal phosphorylation of Tau protein.** *BMC Neurosci* 2008, **9 Suppl 2:**S9.

27. Waskiewicz AJ, Flynn A, Proud CG, Cooper JA: **Mitogen-activated protein kinases activate the serine/threonine kinases Mnk1 and Mnk2.** *EMBO J* 1997, **16:**1909-1920.

28. Qian Z, Lin C, Espinosa R, LeBeau M, Rosner MR: **Cloning and characterization of MST4, a novel Ste20-like kinase.** *J Biol Chem* 2001, **276:**22439-22445.

29. Fry AM, Schultz SJ, Bartek J, Nigg EA: **Substrate specificity and cell cycle regulation of the Nek2 protein kinase, a potential human homolog of the mitotic regulator NIMA of Aspergillus nidulans.** *J Biol Chem* 1995, **270:**12899-12905.

30. Frantz B, Klatt T, Pang M, Parsons J, Rolando A, Williams H, Tocci MJ, O'Keefe SJ, O'Neill EA: **The activation state of p38 mitogen-activated protein kinase determines the efficiency of ATP competition for pyridinylimidazole inhibitor binding.** *Biochemistry* 1998, **37:**13846-13853.

31. Wu H, Wang ZX: **The mechanism of p21-activated kinase 2 autoactivation.** *J Biol Chem* 2003, **278:**41768-41778.

32. Arias-Romero LE, Chernoff J: **A tale of two Paks.** *Biol Cell* 2008, **100:**97-108.

33. Hill MM, Andjelkovic M, Brazil DP, Ferrari S, Fabbro D, Hemmings BA: **Insulin-stimulated protein kinase B phosphorylation on Ser-473 is independent of its activity and occurs through a staurosporine-insensitive kinase.** *J Biol Chem* 2001, **276:**25643-25646.

34. van der Lugt NM, Domen J, Verhoeven E, Linders K, van der Gulden H, Allen J, Berns A: **Proviral tagging in E mu-myc transgenic mice lacking the Pim-1 proto-oncogene leads to compensatory activation of Pim-2.** *EMBO J* 1995, **14:**2536-2544.

35. Hagiwara M, Brindle P, Harootunian A, Armstrong R, Rivier J, Vale W, Tsien R, Montminy MR: **Coupling of hormonal stimulation and transcription via the cyclic AMP-responsive factor CREB is rate limited by nuclear entry of protein kinase A.** *Mol Cell Biol* 1993, **13:**4852-4859.

36. Chen SJ, Klann E, Gower MC, Powell CM, Sessoms JS, Sweatt JD: **Studies with synthetic peptide substrates derived from the neuronal protein neurogranin reveal structural determinants of potency and selectivity for protein kinase C.** *Biochemistry* 1993, **32:**1032-1039.

37. Golsteyn RM, Mundt KE, Fry AM, Nigg EA: **Cell cycle regulation of the activity and subcellular localization of Plk1, a human protein kinase implicated in mitotic spindle function.** *J Cell Biol* 1995, **129:**1617-1628.

38. Force T, Bonventre JV, Heidecker G, Rapp U, Avruch J, Kyriakis JM: **Enzymatic characteristics of the c-Raf-1 protein kinase.** *Proc Natl Acad Sci U S A* 1994, **91:**1270-1274.

39. Doe C, Bentley R, Behm DJ, Lafferty R, Stavenger R, Jung D, Bamford M, Panchal T, Grygielko E, Wright LL, et al: **Novel Rho kinase inhibitors with anti-inflammatory and vasodilatory activities.** *J Pharmacol Exp Ther* 2007, **320:**89-98.

40. Ross H, Armstrong CG, Cohen P: **A non-radioactive method for the assay of many serine/threonine-specific protein kinases.** *Biochem J* 2002, **366:**977-981.

41. Doi J, Takemori H, Lin XZ, Horike N, Katoh Y, Okamoto M: **Salt-inducible kinase represses cAMP-dependent protein kinase-mediated activation of human cholesterol side chain cleavage cytochrome P450 promoter through the CREB basic leucine zipper domain.** *J Biol Chem* 2002, **277:**15629-15637.

42. Cheng HC, Nishio H, Hatase O, Ralph S, Wang JH: **A synthetic peptide derived from p34cdc2 is a specific and efficient substrate of src-family tyrosine kinases.** *J Biol Chem* 1992, **267:**9248-9256.

43. Hutchison M, Berman KS, Cobb MH: **Isolation of TAO1, a protein kinase that activates MEKs in stress-activated protein kinase cascades.** *J Biol Chem* 1998, **273:**28625-28632.

44. Angeles TS, Yang SX, Steffler C, Dionne CA: **Kinetics of trkA tyrosine kinase activity and inhibition by K-252a.** *Arch Biochem Biophys* 1998, **349:**267-274.
